# Sugar-derived oxazolone pseudotetrapeptide as γ-turn inducer and anion-selective transporter

**DOI:** 10.3762/bjoc.15.234

**Published:** 2019-10-14

**Authors:** Sachin S Burade, Sushil V Pawar, Tanmoy Saha, Navanath Kumbhar, Amol S Kotmale, Manzoor Ahmad, Pinaki Talukdar, Dilip D Dhavale

**Affiliations:** 1 Garware Research Center, Department of Chemistry, Savitribai Phule Pune University (formerly University of Pune), Pune 411007, India,; 2 Indian Institute of Science Education and Research, Pune, Pune 411008, India

**Keywords:** ion transport, oxazolone, peptidomimetics, pseudo-peptides, sugar amino acid

## Abstract

The intramolecular cyclization of a C-3-tetrasubstituted furanoid sugar amino acid-derived linear tetrapeptide afforded an oxazolone pseudo-peptide with the formation of an oxazole ring at the *C*-terminus. A conformational study of the oxazolone pseudo-peptide showed intramolecular C=O···HN(II) hydrogen bonding in a seven-membered ring leading to a γ-turn conformation. This fact was supported by a solution-state NMR and molecular modeling studies. The oxazolone pseudotetrapeptide was found to be a better Cl^−^-selective transporter for which an anion–anion antiport mechanism was established.

## Introduction

**Figure 1:**
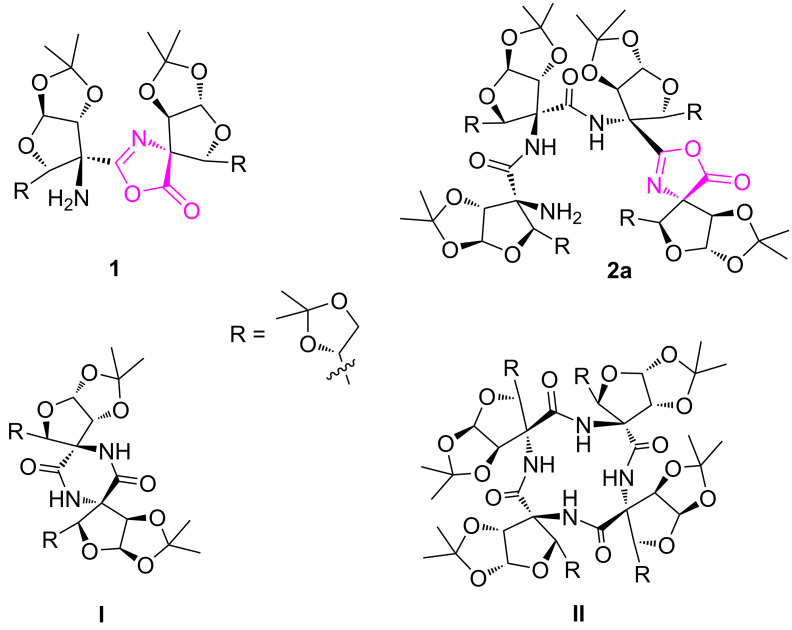
Oxazolone pseudodipeptide **1** and tetrapeptide **2a**.

Tetrasubstituted α-amino acid (TAA)-derived peptidomimetics offer well-defined turn structures due to the presence of a stereochemically stable quaternary carbon center [1]. For example, TAA-derived peptides containing a cyclopropane ring and ʟ/ᴅ-dimethyl tartrate showed an α-turn and form 3_10_-helical conformations in higher oligomers [2–4]. While, TAA-derived peptides having a tetrahydrofuran ring demonstrated a β-turn type conformation [5]. Amongst these, the use of sugar-derived TAAs in peptidomimetics is less explored. The linear tri-/tetrapeptides and spiro-peptides at the anomeric position of mannofructose are known [6–8]. Stick and co-workers have reported the synthesis of tetrasubstituted sugar furanoid amino acid (TSFAA)-derived homologated linear pentapeptide which showed a well defined intramolecular hydrogen-bonding-stabilized helical array [9–11]. Our group has reported a *trans*-vicinal ᴅ-glucofuranoroic-3,4-diacid with a TAA framework and incorporated it into the *N*-terminal tetrapeptide sequence (H-Phe-Trp-Lys-Thy-OH) to get a glycopeptide which acts as an α-turn inducer [12]. Over the last several years, synthetic peptides are known to play a significant role in the design of artificial ion transport systems [13–16]. Recently, our group has synthesized fluorinated acyclic and cyclic peptides from C-3 fluorinated ᴅ-glucofuranoid amino acids and demonstrated their selective anion transport activity [17–18]. In continuation of our interest in sugar-derived cyclic peptides [19], we aimed to synthesize cyclic peptides **I** and **II** from the corresponding linear di- and tetrapeptides, however, we obtained an oxazolone ring containing pseudo peptides **1** and **2a,** respectively (Figure 1) The NMR studies of pseudotetrapeptide **2a** indicated a γ-turn conformation stabilized by the intramolecular hydrogen bonding [(II)NH···O=C] in a seven-membered ring. The oxazolone pseudotetrapeptide **2a** demonstrated better selective Cl^−^ ion transport activity as compared to the pseudodipeptide **1**. To the best of our knowledge, this is the first report on the formation of oxazolone peptides from TSFAA that induces a γ-turn and demonstrate ion transport activity.

## Results and Discussion

**Scheme 1:**
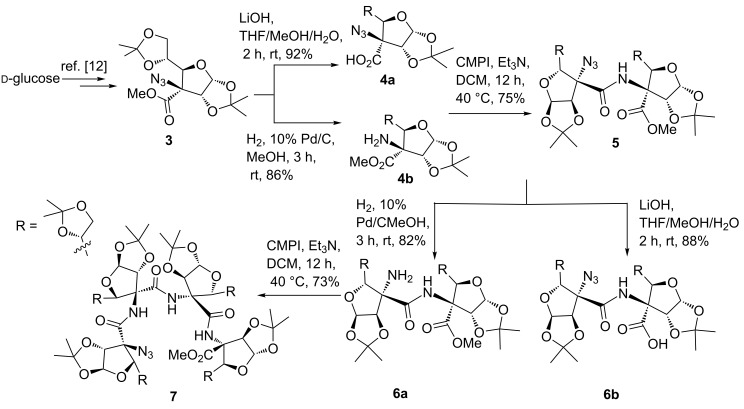
Synthesis of linear azido ester dipeptide **5** and tetrapeptide **7**.

At first, ᴅ-glucose was converted to C-3-tetrasubstituted furanoid sugar azido ester **3** as per our reported protocol [12]. Hydrolysis of the ester functionality in **3** with LiOH at room temperature afforded azido acid **4a** in 92% yield, while hydrogenation of **3** using 10% Pd/C in MeOH at room temperature for 3 h afforded the amino ester **4b** in 86% yield (Scheme 1). The coupling of **4a** and **4b** using 2-chloro-1-*N*-methylpyridinium iodide (CMPI), as a coupling reagent, in the presence of Et_3_N in dichloromethane at 40 °C for 12 h gave azido ester dipeptide **5** in 75% yield. Hydrogenation of **5** using 10% Pd/C in methanol gave amino ester dipeptide **6a** in 82% yield, while hydrolysis of **5** using LiOH gave azido acid dipeptide **6b** in 88% yield. Coupling of **6a** and **6b** using CMPI in the presence of Et_3_N in dichloromethane afforded azido ester tetra-peptide **7** in 73% yield. [20].

**Scheme 2:**
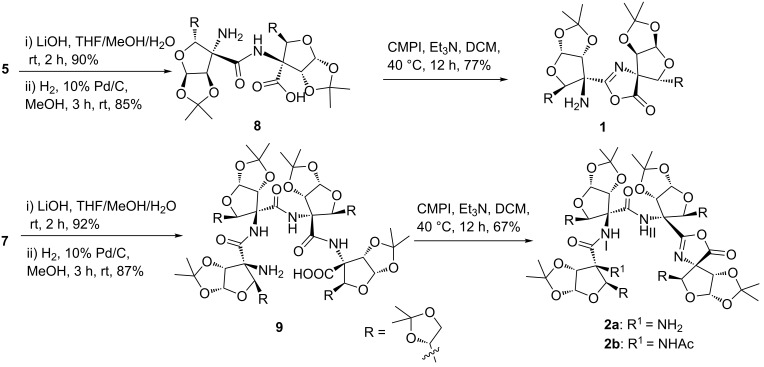
Synthesis of oxazolone pseudopeptides **1**, **2a** and **2b**.

The linear azido ester dipeptide **5** and tetrapeptide **7** were individually converted to amino acid di- and tetrapeptides **8** and **9**, respectively, using hydrolysis followed by a hydrogenation reaction protocol (Scheme 2). In order to get cyclic peptides **I** and **II** (Figure 1), an individual intramolecular coupling reaction of linear dipeptide **8** and tetrapeptide **9** was attempted. Thus, coupling reactions of **8**/**9** with different reagents (HATU, TBTU, PyBOP, EDC·HCl), under a variety of solvents (DMF, acetonitrile, dichloromethane) and reaction conditions (25–80 °C for 24 h) were unsuccessful. This could be due to the stable helical conformation of **8** and **9** in which reactive acid and amino functionalities are apart from each other. However, an individual intramolecular coupling reaction of **8** and **9** using CMPI as a coupling reagent, in the presence of Et_3_N in dichloromethane, afforded pseudodipeptide **1** and pseudotetrapeptide **2a**, respectively, with oxazolone ring formation at the *C*-terminal of the peptides [21–23]. The free amino group in **2a** was acetylated with Ac_2_O/pyridine in dichloromethane to get –NHAc derivative **2b** (Scheme 2).

The single crystal formation of oxazolone psudopeptides **1**, **2a** and **2b** were unsuccessful under a variety of solvent conditions. The ^1^H and ^13^C NMR spectra of **1**, **2a** and **2b** showed sharp and well-resolved signals in CDCl_3_ solution indicating the absence of rotational isomers (Figures S1, S3, and S4 in Supporting Information File 1). The oxazolone pseudodipeptide **1** is devoid of amide linkages and is therefore not considered for conformational studies [24]. In the case of **2a**, the assignment of chemical shifts to different protons was made based on ^1^H,^1^H-COSY, ^1^H,^13^C-HMBC/HSQC, NOESY, and ^1^H,^15^N-HSQC/HMBC studies (Figures S5–S10 in Supporting Information File 1) and values thus obtained are given in Table S1 in Supporting Information File 1. The IR spectrum of **2a** showed a broad band at 3444–3421 cm^−1^ indicating the presence of -NHs of amine/amide functionalities. The bands at 1740 and 1688 cm^−1^ were assigned to the lactone carbonyl and amide (as well as imine) groups, respectively. In the ^1^H NMR spectrum, the downfield signals at δ 9.03 and 8.52 ppm were assigned to the amide NH(I) and NH(II), respectively. The signal at δ 1.80 ppm, integrating for two protons, was assigned to the presence of an NH_2_ functionality. In the ^13^C NMR spectrum, the appearance of signals at δ 170.8, 170.6 and 166.7 ppm were assigned to the lactone/amide carbonyl functionalities. The signal at δ 163.0 ppm was assigned to the -*C*=N functionality. The ^1^H,^15^N-HSQC and ^1^H,^15^N-HMBC spectra showed a signal at δ 246.0 ppm that was assigned to the imine (C=*N*-) nitrogen. The signal at δ 26.2 ppm was assigned to the amine (*N*H_2_) nitrogen. The signals at δ 112.8 and δ 114.1 ppm were due to the nitrogen of amide (CO*N*H) groups. Based on the ^15^N NMR spectra, the presence of the oxazolone ring at the *C*-terminus in **2a** was confirmed [21–23].

The ^1^H NMR spectra of *N*-acylated compound **2b** showed three downfield signals at δ 8.24, 8.19 and 8.09 ppm due to the three amide NHs. An additional singlet at δ 2.0 ppm, integrating for three protons, was assigned to the NHCOC*H*_3_. In the ^13^C NMR spectrum, the appearance of five signals in the downfield region (at δ 171.6, 170.9, 167.5, 165.0, and 164.0 ppm) indicated the presence of three amides, lactone carbonyl and imine carbon (-*C*=N) suggesting the presence of oxazolone ring in **2b**.

### Conformational study of **2a**

**Figure 2:**
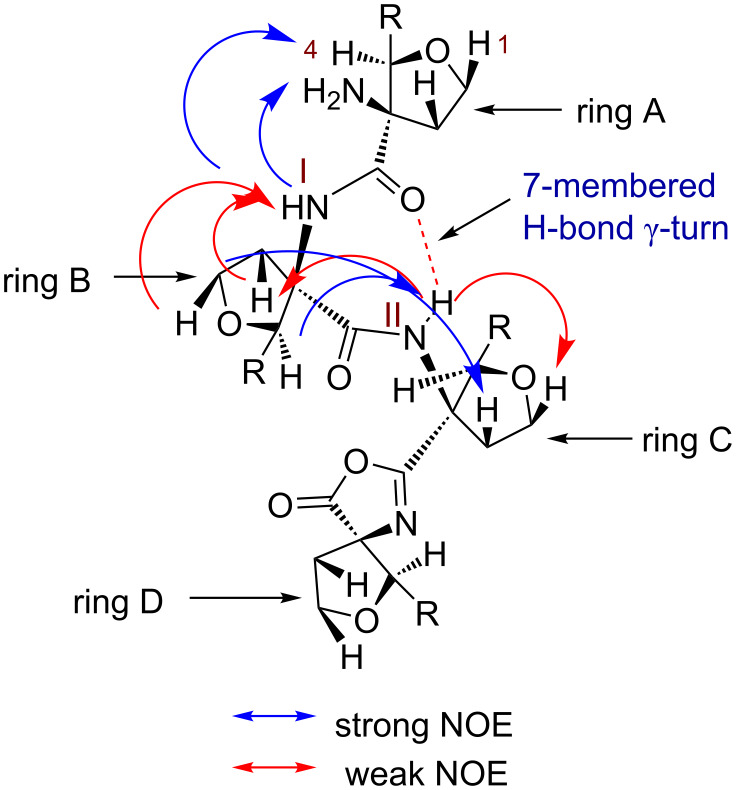
Characteristic NOEs of **2a**.

The downfield shift of amide NH protons δ > 7.5 ppm in **2a** suggested the possible involvement of intramolecular hydrogen bonding [25]. The observed NOESY cross peaks of NH(I)↔NH_2_ indicated closer proximity and orientation on the same side (Figure 2). This is likely to involve (I)NH···NH_2_ weak intramolecular hydrogen bonding. The amide NH(II) showed strong cross peaks with H-2, H-5 of ring C, H-4 of ring B and weak cross peaks with H-1, H-6 of ring C indicating closer proximity and orientation of these protons on the same side. Appearance of strong NOE between NH(II)↔H-4 and weak NOE between NH(II)↔H-2 of ring B indicated the orientation of NH(II) towards the carbonyl group of ring A with the formation of intramolecular hydrogen bonding in a seven-membered ring leading to the γ-turn conformation (Figure 2).

**Figure 3:**
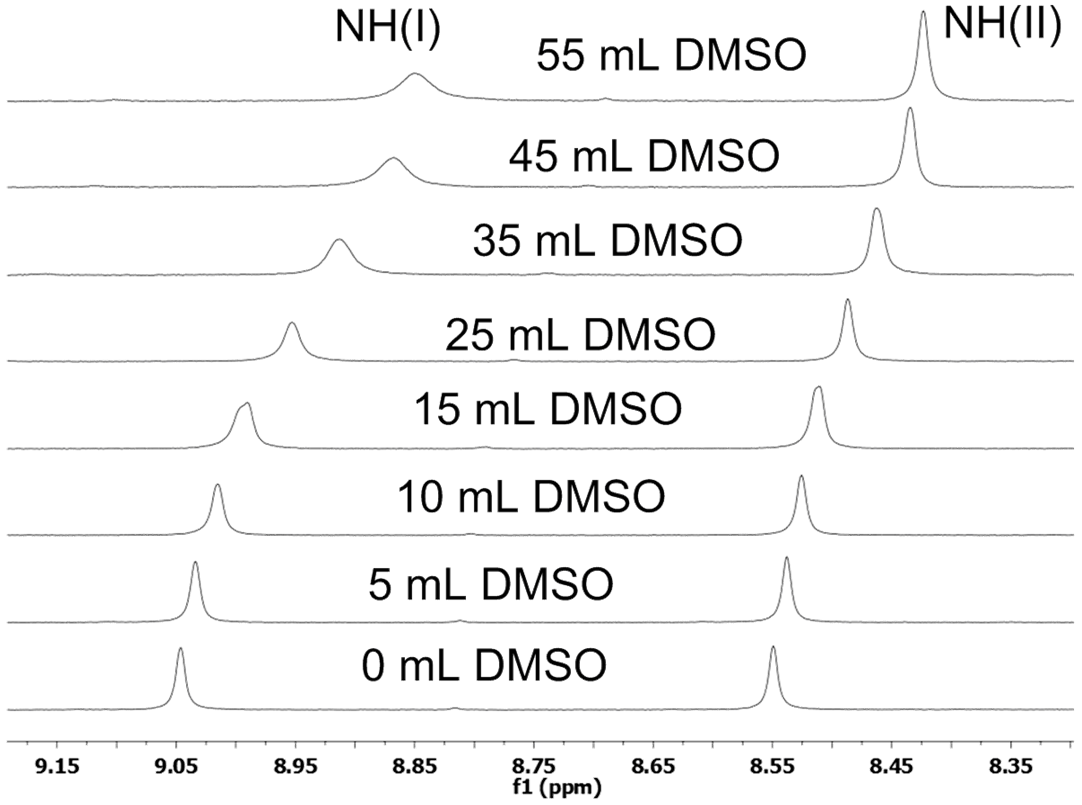
DMSO titration study of **2a**.

The involvement of amide NHs in intramolecular H-bonding was supported by the DMSO-*d*_6_ titration studies. Thus, 5 μL of DMSO-*d*_6_ was sequentially added (up to 50 μL) to the CDCl_3_ solution of **2a** and change in δ value of NH protons was monitored by the ^1^H NMR [26]. The NH(I) proton showed the higher change in chemical shift ∆δ = 0.2 ppm indicating weak (I)NH···NH_2_ intramolecular H-bonding. The NH(II) showed smaller ∆δ = 0.13 ppm suggesting strong (II)NH···O=C intramolecular bonding (Figure 3).

**Figure 4:**
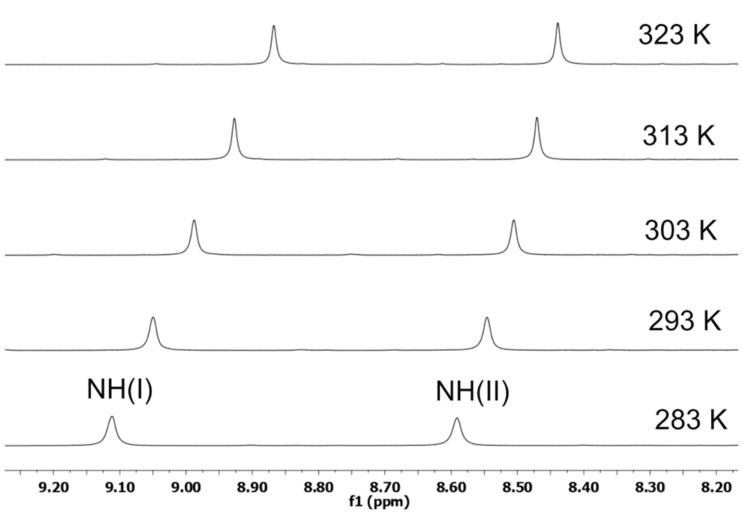
^1^H NMR temperature study of **2a**.

This fact was further supported by a temperature-dependent ^1^H NMR study [27–28]. The temperature-dependent ^1^H NMR of **2a** in CDCl_3_ as solvent at 283–323 K was recorded that showed a higher Δδ/Δ*T* value of 6.2 × 10^−3^ ppm/K for NH(I) indicating its involvement in weak intramolecular H-bonding. For NH(II) the lower Δδ/Δ*T* value of 3.7 × 10^−3^ ppm/K supported its association in strong intramolecular hydrogen bonding with C=O leading to the γ-turn formation (Figure 4).

The ^1^H NMR dilution study of **2a** in CDCl_3_ solution showed the negligible change (∆δ = 0.01) in the chemical shift of NH(I) and(II) protons (Figure S17, Supporting Information File 1), further supporting their intramolecular hydrogen bonding with the free NH_2_ and C=O, respectively. These studies thus supported the presence of γ-turn helical type conformation of **2a**.

### Molecular modeling studies

**Figure 5:**
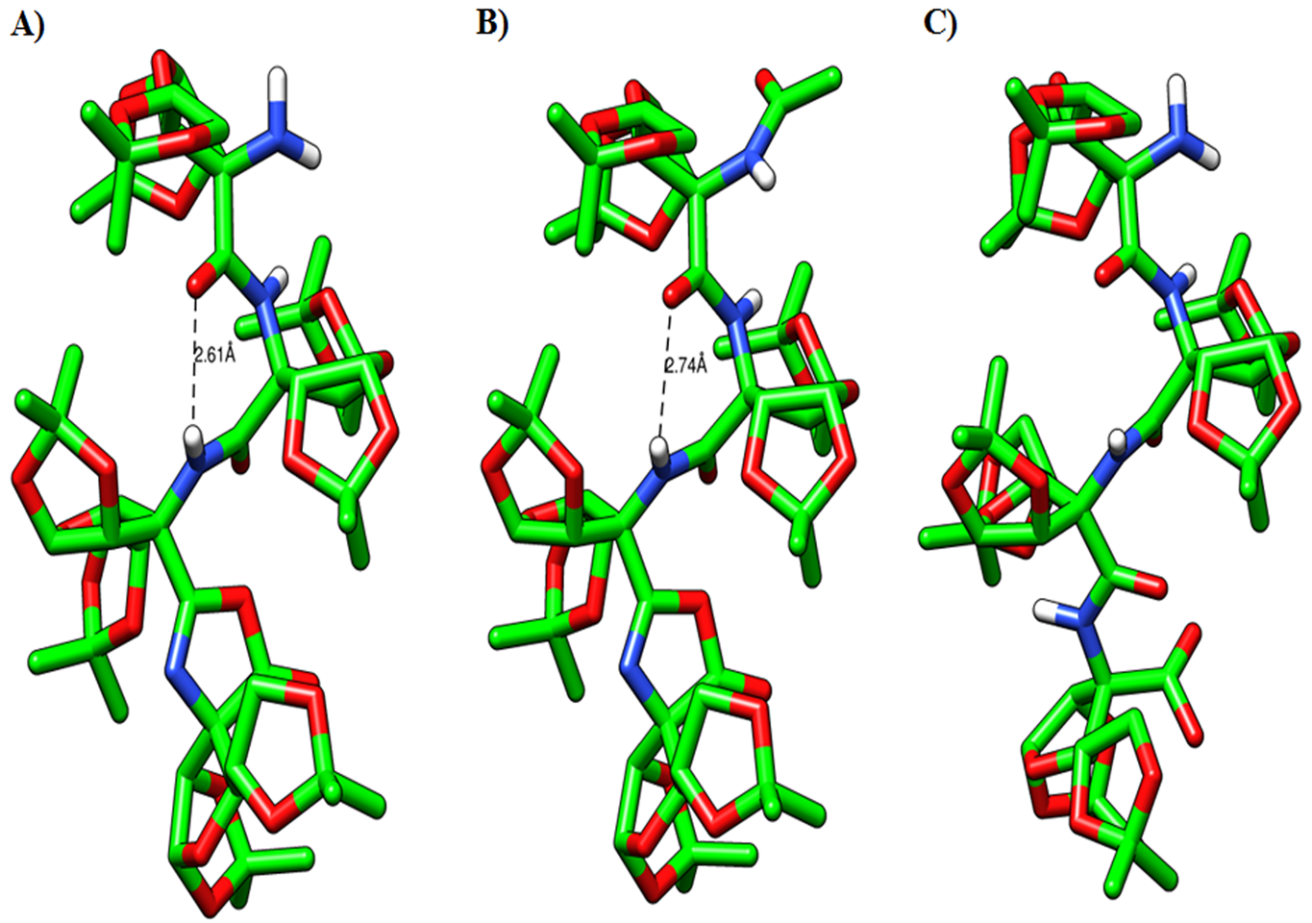
Optimized helical conformations of (A) **2a**, (B) **2b** and (C) **9**.

In order to corroborate our results, obtained from the NMR studies, the molecular modeling study was performed using Spartan’14 software [29–30]. The initial geometry of **2a,** generated from the NOESY study, was subjected to geometry optimization using a semi-empirical PM6 method. The resulted optimized structure of **2a** indicated considerable crowding due to the presence of the oxazolone ring and two acetonide rings of sugar ring D (Figure 5A). To accumulate the oxazolone ring, the sugar ring C is pushed towards the A and B rings. The γ-turn conformation is stabilized by the intramolecular (II)NH···O=C hydrogen bonding in a seven-membered ring [bond distance (*d*) = 2.61 Å and bond angle (NH···O) = 114.06°]. To understand the role of the oxazolone ring in stabilizing the γ-turn, we performed geometry optimization on TFSAA linear tetrapeptide amino acid **9** (Figure 5C). The optimized geometry of **9** showed a change in helical conformation overcome the crowding due to acetonide groups. The *N*- and *C*-terminals are further away, thus precluding the γ-turn conformation [(bond distance (*d*) = 3.11 Å) and bond angle (NH···O) = 98.90°]. The comparison of geometrically optimized models of **2a** and **9** showed small structural changes with respect to the helical pitch length. The distance between C=O···N(II) is 3.18 Å in **2a** and 3.43 Å in **9**. The distance between Cα1···Cα4 is 9.67 Å in **2a** and 9.84 Å in **9** (Figure S18 in Supporting Information File 1). Similarly, the distance between N1···C4 is 9.44 Å in **2a** and 10.47 Å in **9**. This suggested an elongated helical structure of linear tetrapeptide **9** than **2a**. Thus, the compact helical architecture of **2a** is due to the presence of the oxazolone ring leading to a γ-turn conformation. The molecular modeling study of *N*-acetylated compound **2b** also indicated the presence of a seven-membered hydrogen bonding between NH(II) and –C=O [bond distance (*d*) = 2.74 Å and bond angle (NH···O) = 112.98°] suggesting the presence of a γ-turn conformation (Figure 5B).

### Ion transport activity

The cation and anion transport across lipid bilayer membranes plays a crucial role in various biological processes [31–32]. Amongst these, the transport of anions is useful in regulating intracellular pH, membrane potential, cell volume, and fluid transport [33]. Any dysfunction in these processes led to various diseases such as cystic fibrosis, Dent disease, Bartter syndrome, and epilepsy [34–37]. In order to mimic the regulatory functions in living systems, a wide range of anion transporters have been investigated that include peptides [38–43], oligoureas [44–45], anion-π slides [46–47], steroids [48–49], calixpyrroles [50–51], calixarenes [52–53], and other scaffolds [54–56]. In particular, peptide based transmembrane anion transporters have attracted great interest. For example, Ghadiri [38], Ranganathan [39], and Granja [40] have independently reported different types of cyclic peptides as anion transporters. Gale, Luis, and co-workers [41] have separately reported the linear pseudopeptides as receptors and transporters of chloride and nitrate anions.

**Figure 6:**
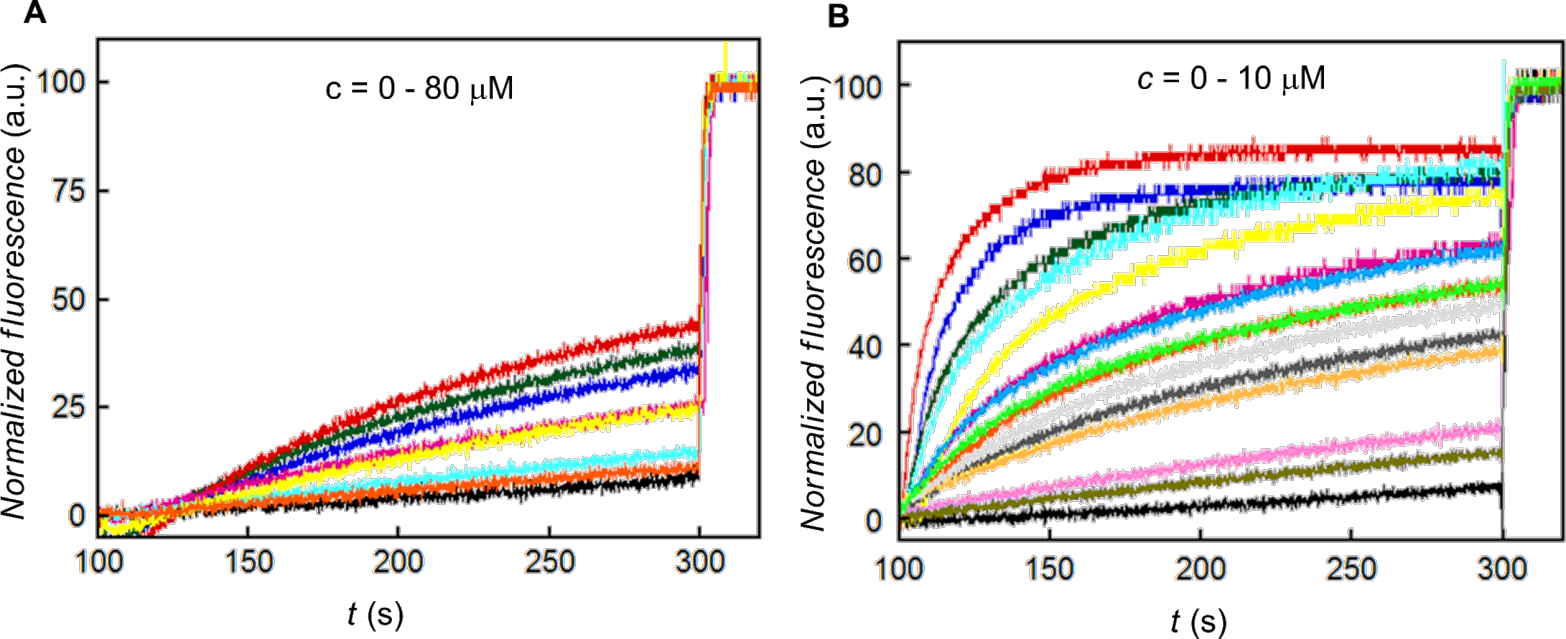
Ion transport activity (A) for **1**, (B) for **2a**, across EYPC-LUVs HPTS.

Inspired by our recent ion transport studies with fluorinated acyclic and cyclic sugar derived peptides [17–18], we investigated the ion transport activity of **1** and **2a** across lipid bilayer membranes. In this study, the collapse of the pH gradient (pH_out_ = 7.8 and pH_in_ = 7.0), created across egg yolk ʟ-α-phosphatidylcholine (EYPC) vesicles with entrapped 8-hydroxypyrene-1,3,6-trisulfonic acid trisodium salt (HPTS) dye (i.e., EYPC-LUVs
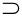
HPTS) [57–61] was monitored by measuring the fluorescence intensity of the dye at λ_em_ = 510 nm (λ_ex_ = 450 nm) with time (Figure S11, Supporting Information File 1). Thus, the addition of **2a** (10 µM) resulted in the significant increase in HPTS fluorescence within 200 s (Figure 6B), while oxazolone pseudodipeptide **1** was found to be lesser active (Figure 6A).

**Figure 7:**
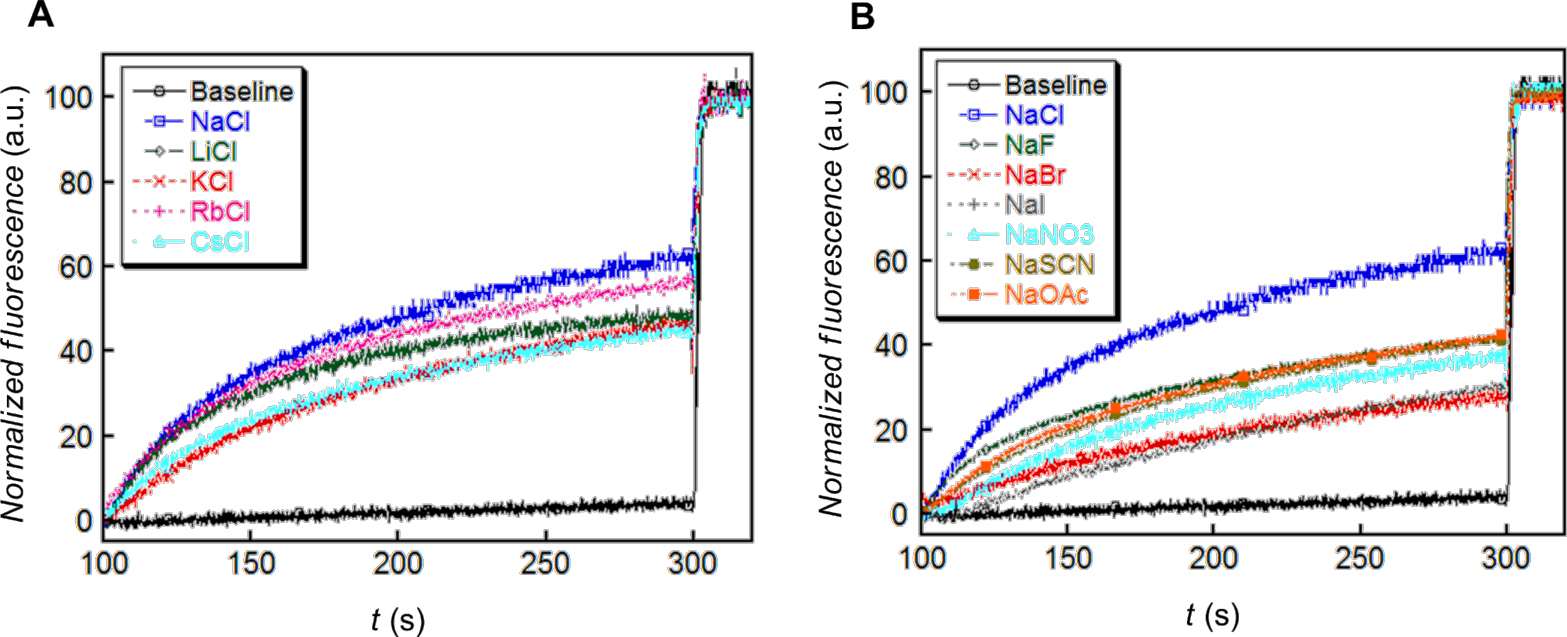
Cation (A) and anion (B) transport activity of **2a**.

From the dose–response data of **2a**, the calculated effective concentration *EC*_50_ = 0.72 µM indicated good ion transport activity of **2a** (Figure S12 in Supporting Information File 1). The Hill coefficient *n* value of 1.26 indicated that one molecule of **2a** is involved in the formation of the active transporter. The promising ion transport activity of **2a** encouraged us to explore its cation and anion selectivity study by varying either cations (for MCl, M^+^ = Li^+^, Na^+^, K^+^, Rb^+^, and Cs^+^) or anions (for NaA, A^−^ = F^−^, Cl^−^, Br^−^, I^−^, NO_3_^−^, SCN^−^, AcO^−^ and ClO_4_^−^) of the extravesicular salt, respectively. Thus, variation of external cations, in the presence of **2a** (0–10 µM), showed minor changes in the transport activity with the sequence: Na^+^ > Rb^+^ > Li^+^ > K^+^ ≈ Cs^+^ (Figure 7A), which suggest lesser influence of alkali metal cations in the transport process. However, variation of extravesicular anions demonstrated the changes in the transport behaviour with the following selectivity sequence: Cl^–^ >> AcO^–^ ≈ SCN^–^ ≈ F^–^ > NO_3_^–^ >> Br^–^ ≈ I^–^, showing highest selectivity for the Cl^–^ ion (Figure 7B). Overall, anion variation had more influence in the ion transport rate compared to the cations.

### Chloride leakage study

**Figure 8:**
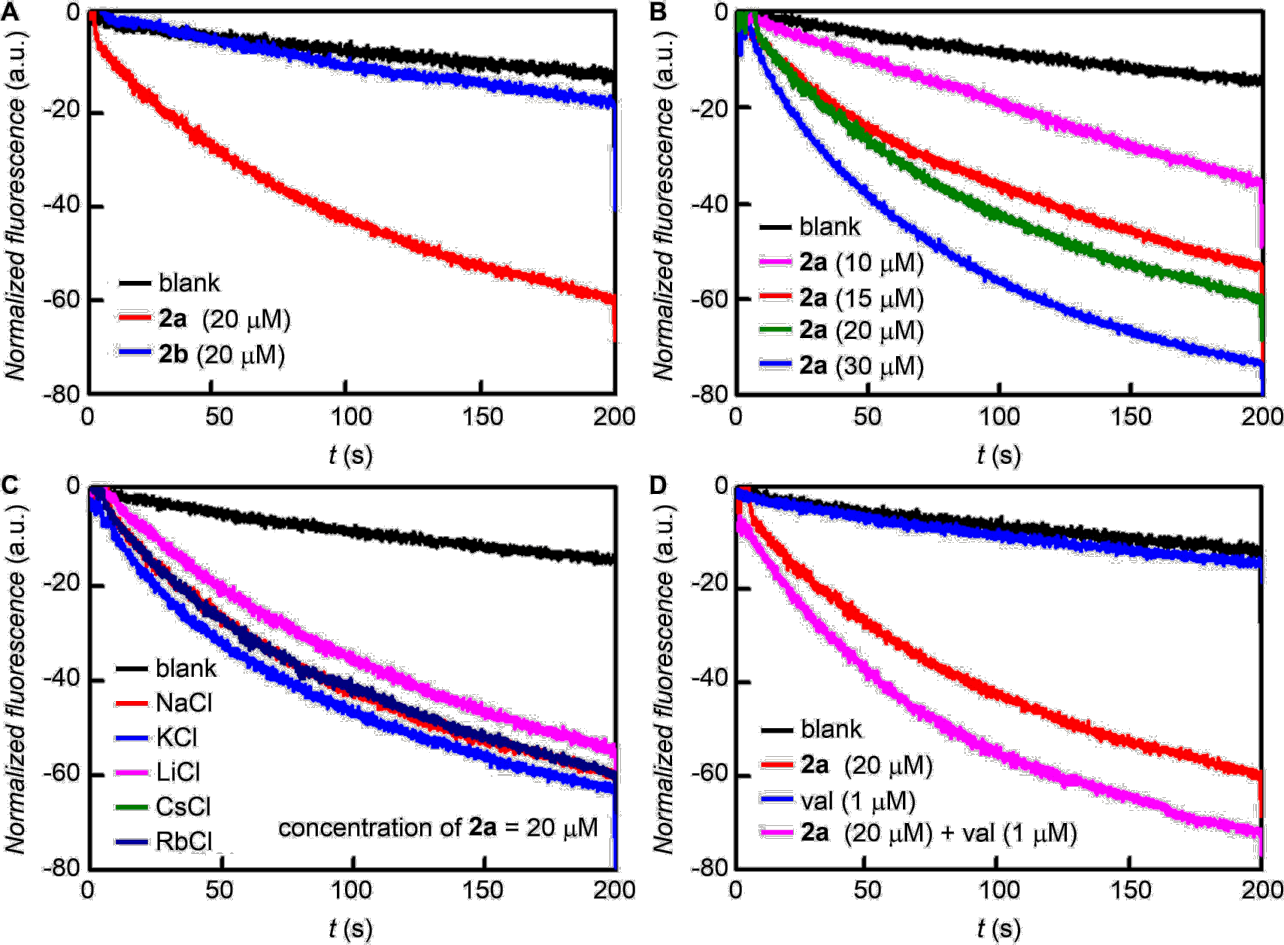
Comparison of the ion transport activity of **2a** and **2b** at 20 µM across EYPC-LUVs
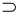
lucigenin (A). Concentration dependent activity of **2a** across EYPC-LUVs
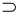
lucigenin (B). Transport activity of **2a** (20 µM) by changing extravesicular cations (C). Transport activity of **2a** (20 µM) in the presence and absence of valinomycin (1 µM) across EYPC-LUVs
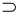
lucigenin (D).

In order to know the role of the free NH_2_ group in **2a** for Cl^−^ recognition during the transport of the ion, we monitored the Cl^−^ transport activities of the amino compound **2a** and its *N*-acylated derivative **2b**. The influx of Cl^‒^ ion by these transporters were monitored using EYPC-LUVs
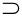
lucigenin. Additionally, compound **9**, which has a free amino group and a free carboxylic acid group, was also subjected to the Cl^‒^ transport study. The Cl^–^ sensitive dye lucigenin, was entrapped within the lipid vesicles and the rate of quenching in fluorescence at λ_em_ = 535 nm (λ_ex_ = 455 nm) was monitored using transporter **2a** by creating a Cl^–^ gradient across the lipid membrane by applying NaCl in the extravesicular buffer (Figure S14 in Supporting Information File 1). The compound **2a** showed a significant decrease in the fluorescence rate of lucigenin and the change in fluorescence upon the addition of **2a** (Figure 8A and 8B). We observed that the *N*-acetylated compound **2b** was observed to be inactive (Figure 8A), indicating that the free amine group is necessary for the transport activity. Compound **9** did not exhibit any transport activity even at very high concentration (Figure S16 in Supporting Information File 1).

Further, the variation of cations in the extravesicular buffer using different salts of MCl (M^+^ = Li^+^, Na^+^, K^+^, Rb^+^, and Cs^+^) does not make any change in the transport rate of **2a** (20 µM) which excludes any role of cation in an overall transport process (Figure 8C). Finally, to evaluate the mechanism of ion transport, the transport of Cl^–^ using compound **2a** (20 µM) was monitored in the presence and absence of valinomycin (a selective K^+^ transporter, 1 μM). There was a significant increase in the transport rate of **2a** in the presence of valinomycin confirming the transport process occurring through an antiport mechanism via Cl^−^/NO_3_^−^ exchange (Figure 8D). Such anion-selective transport can be rationalized based on the binding of anions with the terminal amino group of the transporter through hydrogen bond interaction. However, the role of the neighboring amido groups cannot be ruled out. Moreover, the hydrophobic outer surface of the transporter helps the anion bound complex to permeate efficiently through the lipid bilayer membranes.

## Conclusion

In conclusion, the intramolecular cyclization of linear di- and tetrapeptides **8** and **9** led to the formation of the oxazolone ring at the C-terminal giving pseudopeptides **1** and **2a**, respectively. The pseudotetrapeptide **2a** showed a γ-turn conformation that is stabilized by a seven-membered intramolecular hydrogen bonding. The pseudotetrapeptide **2a** was found to facilitate selective anion transport that occurs by an anion–anion antiport mechanism. The absence of the γ-turn conformation as well as ion transport activity in linear tetrapeptide **9** – the precursor of **2a**, suggest that the oxazolone ring in **2a** is a γ-turn inducer as well as responsive for selective anion transport activity.

## Supporting Information

File 1 Experimental procedures, ^1^H and ^13^C NMR data, HRMS and 2D NMR spectra.

## References

[1] Maity P, Konig B (2008). Pept Sci.

[2] Bonora G M, Toniolo C, Di Blasio B, Pavone V, Pedone C, Benedetti E, Lingham I, Hardy P (1984). J Am Chem Soc.

[3] Toniolo C, Bonora G M, Barone V, Bavoso A, Benedetti E, Di Blasio B, Grimaldi P, Lelj F, Pavone V, Pedone C (1985). Macromolecules.

[4] Demizu Y, Doi M, Kurihara M, Maruyama T, Suemune H, Tanaka M (2012). Chem – Eur J.

[5] Maity P, Zabel M, König B (2007). J Org Chem.

[6] Estevez J C, Estevez R J, Ardron H, Wormald M R, Brown D, Fleet G W J (1994). Tetrahedron Lett.

[7] Estevez J C, Ardron H, Wormald M R, Brown D, Fleet G W J (1994). Tetrahedron Lett.

[8] Estevez J C, Smith M D, Wormald M R, Besra G S, Brennan P J, Nash R J, Fleet G W J (1996). Tetrahedron: Asymmetry.

[9] Forman G S, Scaffidi A, Stick R V (2004). Aust J Chem.

[10] Scaffidi A, Skelton B W, Stick R V, White A H (2007). Aust J Chem.

[11] Scaffidi A, Skelton B W, Stick R V, White A H (2004). Aust J Chem.

[12] Vangala M, Dhokale S A, Gawade R L, Pattuparambil R R, Puranik V G, Dhavale D D (2013). Org Biomol Chem.

[13] Djedovic N, Ferdani R, Harder E, Pajewska J, Pajewski R, Weber M E, Schlesinger P H, Gokel G W (2005). New J Chem.

[14] García-Fandiño R, Amorín M, Castedo L, Granja J R (2012). Chem Sci.

[15] Sakai N, Houdebert D, Matile S (2003). Chem – Eur J.

[16] Zeng F, Liu F, Yuan L, Zhou S, Shen J, Li N, Ren H, Zeng H (2019). Org Lett.

[17] Burade S S, Shinde S V, Bhuma N, Kumbhar N, Kotmale A, Rajamohanan P R, Gonnade R G, Talukdar P, Dhavale D D (2017). J Org Chem.

[18] Burade S S, Saha T, Bhuma N, Kumbhar N, Kotmale A, Rajamohanan P R, Gonnade R G, Talukdar P, Dhavale D D (2017). Org Lett.

[19] Pawar N J, Diederichsen U, Dhavale D D (2015). Org Biomol Chem.

[R20] 20Synthesis of azido dipeptide **5** and tetrapeptide **7** is reported [11] using TsCl in pyridine as an activating agent for the carboxylic group. The same reaction at our hand gave the dark brown coloured product that on purification afforded ≈30% yield while; the use of CMPI as coupling reagent gave pale yellow solid product that on purification gave ≈75% yield of **5** and **7**.

[21] King S W, Stammer C H (1981). J Org Chem.

[22] Yagisawa S, Urakami M (1996). Tetrahedron Lett.

[23] Sakamoto S, Kazumi N, Kobayashi Y, Tsukano C, Takemoto Y (2014). Org Lett.

[R24] 24Further, reactions of **1** and **2** under different acidic/basic conditions gave complex mixture of products thus precluding extension of work.

[25] Nowick J S, Smith E M, Pairish M (1996). Chem Soc Rev.

[26] El-Faham A, Albericio F (2011). Chem Rev.

[27] Kishore R, Kumar A, Balaram P (1985). J Am Chem Soc.

[28] Gellman S H, Dado G P, Liang G B, Adams B R (1991). J Am Chem Soc.

[29] Hehre W J, Radom L, Schleyer P v R (1986). Ab Initio Molecular Orbital Theory.

[30] Stewart J J P (2007). J Mol Model.

[31] Hille B (2001). Ion Channels of Excitable Membranes.

[32] Benz R, Hancock R E W (1987). J Gen Physiol.

[33] Beer P D, Gale P A (2001). Angew Chem, Int Ed.

[34] Bitter E E, Pusch M (2006). Chloride movements across cellular membranes.

[35] Jentsch J J T, Stein V, Weinrich F, Zdebik A A (2002). Physiol Rev.

[36] Busschaert N, Gale P A (2013). Angew Chem, Int Ed.

[37] Choi J Y, Muallem D, Kiselyov K, Lee M G, Thomas P J, Muallem S (2001). Nature.

[38] Bong D T, Clark T D, Granja J R, Ghadiri M R (2001). Angew Chem, Int Ed.

[39] Ranganathan D (2001). Acc Chem Res.

[40] Brea R J, Reiriz C, Granja J R (2010). Chem Soc Rev.

[41] Martí I, Burguete M I, Gale P A, Luis S V (2015). Eur J Org Chem.

[42] Schlesinger P H, Ferdani R, Liu J, Pajewska J, Pajewski R, Saito M, Shabany H, Gokel G W (2002). J Am Chem Soc.

[43] Benke B P, Madhavan N (2013). Chem Commun.

[44] Diemer V, Fischer L, Kauffmann B, Guichard G (2016). Chem – Eur J.

[45] Li A-F, Wang J-H, Wang F, Jiang Y-B (2010). Chem Soc Rev.

[46] Gorteau V, Bollot G, Mareda J, Perez-Velasco A, Matile S (2006). J Am Chem Soc.

[47] Gorteau V, Julliard M D, Matile S (2008). J Membr Sci.

[48] McNally B A, Koulov A V, Smith B D, Joos J-B, Davis A P (2005). Chem Commun.

[49] Hussain S, Brotherhood P R, Judd L W, Davis A P (2011). J Am Chem Soc.

[50] Fisher M G, Gale P A, Hiscock J R, Hursthouse M B, Light M E, Schmidtchen F P, Tong C C (2009). Chem Commun.

[51] Gale P A, Tong C C, Haynes C J E, Adeosun O, Gross D E, Karnas E, Sedenberg E M, Quesada R, Sessler J L (2010). J Am Chem Soc.

[52] Sidorov V, Kotch F W, Abdrakhmanova G, Mizani R, Fettinger J C, Davis J T (2002). J Am Chem Soc.

[53] Maulucci N, Izzo I, Licen S, Maulucci N, Autore G, Marzocco S, TecillaDe P, De Riccardis F (2008). Chem Commun.

[54] Davis J T, Okunola O, Quesada R (2010). Chem Soc Rev.

[55] Brotherhood P R, Davis A P (2010). Chem Soc Rev.

[56] Gale P A (2011). Acc Chem Res.

[57] Madhavan N, Robert E C, Gin M S (2005). Angew Chem, Int Ed.

[58] Saha T, Dasari S, Tewari D, Prathap A, Sureshan K M, Bera A K, Mukherjee A, Talukdar P (2014). J Am Chem Soc.

[59] Kelly T R, Kim M H (1994). J Am Chem Soc.

[60] Dias C M, Li H, Valkenier H, Karagiannidis L E, Gale P A, Sheppard D N, Davis A P (2018). Org Biomol Chem.

[61] Salunke S B, Malla J A, Talukdar P (2019). Angew Chem, Int Ed.

