# Supplementary material for: Sugar-derived oxazolone pseudotetrapeptide as γ-turn inducer and anion-selective transporter
Source: Beilstein J Org Chem. 2019 Oct 14;15:2419–27. doi: 10.3762/bjoc.15.234 (PMC6808195; doi:10.3762/bjoc.15.234)
Supplement: File 1 — Experimental procedures, 1H and 13C NMR data, HRMS and 2D NMR spectra. [file Beilstein_J_Org_Chem-15-2419-s001.pdf]

BEILSTEIN JOURNAL OF ORGANIC CHEMISTRY

Supporting Information

for

Sugar-derived oxazolone pseudotetrapeptide as γ-turn inducer and anion-selective transporter

Sachin S. Burade, Sushil V. Pawar, Tanmoy Saha, Navanath Kumbhar, Amol S. Kotmale, Manzoor Ahmad, Pinaki Talukdar and Dilip D. Dhavale

Beilstein J. Org. Chem. **2019,** *15,* 2419–2427. doi:10.3762/bjoc.15.234

Experimental procedures, ^{1}H and ^{13}C NMR data, HRMS and 2D NMR spectra

License and Terms: This is a supporting information file under the terms of the Creative Commons Attribution License (http://creativecommons.org/licenses/by/4.0). Please note that the reuse, redistribution and reproduction in particular requires that the authors and source are credited.
The license is subject to the *Beilstein Journal of Organic Chemistry* terms and conditions: (https://www.beilstein-journals.org/bjoc)

Table of contents

Contents	Page numbers
General methods and experimental procedures	S2–S8
^{1}H and ^{13}C NMR spectra	S9–S13
2D-NMR spectra of (COSY, NOESY, ^{15}N HMBC)	S14–S18
Ion transport activity studies	S19–S26
Dilution study of **2a**	S26
Geometrically optimized models of **2a**, **2b** and **9**	S26–S27
References	S27–S28

General methods

All reactions were carried out with distilled and dried solvents using oven-dried glassware. ^{1}H NMR (500 MHz) and ^{13}C NMR (125 MHz) and COSY, NOESY (500 MHz) were recorded in $CDCl_3$. Chemical shifts are reported in δ units (parts per million) with reference to TMS as an internal standard (including DMSO titration study, variable temperature, and dilution studies). Melting points are uncorrected. Optical rotations were measured on a digital polarimeter with sodium light (589.3 nm) at 25 °C. High-resolution mass spectra (HRMS) were obtained in positive ion-electron spray ionization (ESI) mode using a TOF (time of flight) analyzer. Thin-layer chromatography was performed on pre-coated plates (0.25 mm, silica gel 60 F254). Column chromatography was carried out with silica gel (100–200 mesh). IR spectra were recorded on a FTIR spectrophotometer as a thin film or using KBr pellets and reported in cm^{-1}. After neutralization, workup involves washing of the combined organic layer with aqueous sodium bicarbonate, water, brine, drying over anhydrous sodium sulphate and evaporation of solvent under reduced pressure.

Experimental procedures:

Synthesis of 3-deoxy-3β-azido-1,2;5,6-di-*O*-isopropylidene-3α-C-methoxycarbonyl- α-D-glucofuranose, **3** was carried out as per our reported procedure (ref.1).

General rocedure for hydrolysis of methyl ester:

The methyl ester (1 equiv.) was dissolved in THF/MeOH/H_2O (3:1:1) mixture (100 mM). The solution was stirred at 0 °C for 5 min. LiOH (2.5 equiv) was added slowly and the mixture was stirred vigorously at rt for 2 h. The solvent was concentrated under vacuum and diluted with water and acidified to approx. pH 4 with 2 N HCl solution. The aqueous layer was extracted with ethyl acetate (three times). The combined organic layer was dried over Na_2SO_4. The solvent was removed in vacuo to give the corresponding acid.

General rocedure for the conversion of azide to amine:

Azide compound (1 equiv.) was subjected for the reduction in presence of H_2, 10% Pd/C (cat.) in methanol at rt for 3 h. The reaction mixture was filtered through a Celite bed. Organic solvent was concentrated under vacuum to give corresponding free amino compounds.

General rocedure for eptide coupling reaction:

To the stirred solution of amine compound (1 mmol), Et_3N (2 mmol) in dry dichloromethane under nitrogen atmosphere, acid compound (1 mmol) was added at 0 ^{o}C and stirred for 5 min; then CMPI (1 mmol) was added at the same temperature. After 5 min the reaction was refluxed at 40 °C and continued for 12 h. Reaction mixture was diluted with EtOAc (100 mL) and the organic layer was washed with 1 M HCl (50 mL) and saturated aqueous $NaHCO_3$ (50 mL) and brine. The organic layer was dried over Na_2SO_4. The solvent was removed in vacuo to give the crude product of peptide which was purified by column chromatography.

The above procedure was used to synthesize **4a**, **4b**, TSFAA dipeptide **5**, **6a**, **6b** and TSFAA tetrapeptide **7**. The analytical and spectroscopic data of **5** and **7** was found to be identical to that reported (ref 2).

Oxazolone pseudodipeptide **1**:

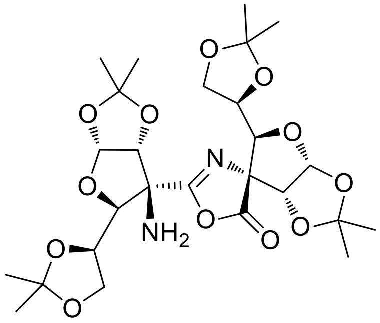

The dipeptide **5** (0.5 g, 0.795 mmol) was dissolved in 2 mL of THF/MeOH/H_2O (3:1:1) and LiOH (0.085 g, 2.04 mmol) was added slowly at room temperature with continuous stirring for 3 h, with the completion of reaction mixture was concentrated in vacuo and the residual solution was acidified by using 1 N HCl solution. The generated acid was extracted with ethyl acetate (3 × 10 mL). The combined organic layers were dried over sodium sulphate, filtered and concentrated in vacuo to give 0.43 g (87.9%) azido-acid dipeptide. The resulting azido-acid

dipeptide (0.43 g, 0.699 mmol, crude) was subjected for hydrogenation in the presence of H_2, 10% Pd/C in methanol with continuous stirring at room temperature for 3h. After completion of reaction the reaction mixture was filtered through celite bed using sintered funnel. The resulting solution of methanol was concentrated in vacuo to get crude amino-acid dipeptide **8** (0.40 g, 0.679 mmol) as a white solid. The crude amine-acid dipeptides **8** (0.40 g, 0.679 mmol) were dissolved in dry dichloromethane with high dilution (≈0.005 M) under inert atmosphere (nitrogen gas), Et_3N (0.137 g, 1.35 mmol, 2 equiv) and CMPI (0.173 g, 0.679 mmol, 1 equiv) was added at rt. Reaction mixture was stirred for 5 h at 40 °C. Reaction mixture was diluted with EtOAc (20 mL) and the organic layer was washed with 1 M HCl (aq) (10 mL) and saturated aqueous $NaHCO_3$ (10 mL) and brine. The organic layer was dried over Na_2SO_4. The solvent was removed in vacuo to give the crude product that was purified by column chromatography to afford oxazolone pseudodipeptide **1** as a white solid (0.3 g ,77%), R_f = 0.45 (hexane/ethyl acetate 1:1), $[\alpha]_D^{25}$ = +48.66 (*c* = 0.11, MeOH), Mp = decomposes to >250 °C, ν_{max} (KBr) = 3394, 3350, 1720, 1686, 1609 cm^{-1}.

^1H NMR (500 MHz, $CDCl_3$): δ = 6.10 (d, *J*= 3.6 Hz, 1H, C_1H^a), 6.01 (d, *J*= 3.6 Hz, 1H, C_1H^b), 4.63 (d, *J*= 8.1 Hz, 1H, C_4H^a), 4.41 (d, *J*= 3.6 Hz, 1H, C_2H^b), 4.40 (d, *J*= 8.7 Hz, 1H, C_4H^b), 4.38 (d, *J*= 3.6 Hz, 1H, C_2H^a), 4.23 - 4.16 (m, 2H, $C_5H^{a,b}$), 4.08 – 3.94 (m, 4H, $C_6H^{a,b}$), 1.8 (br s, 2H, NH_2), 1.65 (s, 3H, CH_3), 1.53 (s, 3H, CH_3), 1.35 (s, 3H, CH_3), 1.32 (s, 3H, CH_3), 1.29 (s, 3H, CH_3), 1.28 (s, 3H, CH_3), 1.24 (s, 3H, CH_3), 1.21 (s, 3H, CH_3).

^{13}C NMR (125 MHz, $CDCl_3$): δ = 171.8 (COO-), 166.5 (-C=N-), 114.1 ($CH_3\underline{C}OOCH_3$-b), 113.2 ($CH_3\underline{C}OOCH_3$-a), 109.7 ($CH_3\underline{C}OOCH_3$-a), 109.7 ($CH_3\underline{C}OOCH_3$-b), 106.8 (C1-b) , 105.5 (C1-a), 88.3 (C2-a), 85.8 (C2-b), 84.5 (C4-b), 81.2 (C4-a), 75.6 (C3-b), 73.3 (C5-b), 73.0 (C5-a), 67.9 (C6-a), 67.3 (C6-b), 64.6 (C3-a), 29.7 (CH_3), 26.9 (CH_3),26.8 (CH_3), 26.8 (CH_3), 26.2 (CH_3), 26.1 (CH_3), 25.2 (CH_3), 25.0 (CH_3).

^{15}N NMR (50 MHz, $CDCl_3$): δ = 27.8 (NH_2), 244 (-C=N-).

HRMS (ESI$^+$): Calculated for $C_{26}H_{39}N_2O_{12}$ (M+H$^+$) 570.2425 found 570.2410.

Tetrapeptide-amino acid compound **9:**

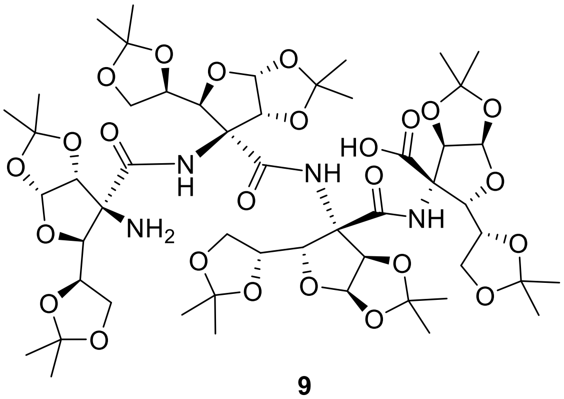

9

^{1}H NMR (400 MHz, $CDCl_3$) δ 8.72 (bs, 1H, NH), 8.51 (bs, 1H, NH), 8.00 (s, 1H, NH), 6.05 (bd, *J* = 2.4 Hz, 4H, H-1), 6.01 (bs, 1H, OH), 5.17 (bd, *J* = 2.4 Hz, 4H, H-2), 4.94 (bs, 2H, H-4), 4.76 (d, *J* = 7.3 Hz, 2H, H-4), 4.45 - 3.79 (m, 14H, H-5, H-6, NH_2), 1.65 – 1.28 (m, 48H, $-CH_3$).

^{13}C NMR (100 MHz, $CDCl_3$) δ 167.6, 167.5, 166.8, 166.7, 113.8, 113.4, 113.1, 112.9, 110.3, 110.1, 109.9, 106.8, 105.5, 84.1, 83.9, 82.8, 82.6, 73.70, 73.5, 72.1, 71.9, 70.3, 70.1, 67.9, 67.7, 67.3, 66.8, 26.9, 26.8, 26.7, 26.5, 26.3, 26.1, 25.7, 25.5.

$[\alpha]^{25}_{D}$ = +185.40 (*c* = 0.1, $CHCl_3$), Mp = decomposes to >243 °C, ν_{max} (KBr) = 3510, 3331, 2967, 2937, 2905, 1679, 1510, 1376, 1073 cm^{-1}.

Oxazolone pseudotetrapeptide **2a**:

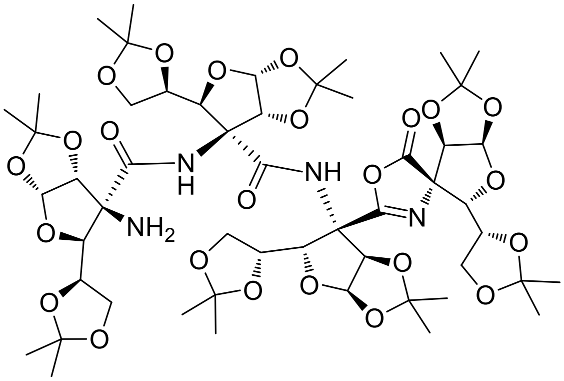

The tetrapeptide **7** (0.5g, 0.4171 mmol) was dissolved in 2 mL of THF/MeOH/H_2O (3:1:1) and LiOH (0.042 g, 1.04 mmol) was added slowly at room temperature with continuous stirring for 3h, with the completion of reaction methanol was concentrated in vacuo and the residual solution was acidified by using 1 N HCl. The generated acid was extracted with ethyl acetate (3 × 10 mL). The combined organic layers were dried over sodium sulphate, filtered and concentrated in vacuo to give 0.45 g (92%) of the azido-acid tetrapeptide. The resulting azido-acid tetrapeptide (0.45 g, 0.379 mmol, crude) was subjected for hydrogenation in presence of H_2, 10% Pd/C in methanol with continuous stirring at room temperature for 3h. After completion of reaction the reaction mixture was filtered through celite using sintered funnel. The resulting solution of methanol was concentrated in vacuo to get crude amino-acid tetrapeptide **9** as foamy solid (0.44 g, 0.379 mmol). The crude amine-acid tetrapeptides **9** (0.44 g, 0.379 mmol) were dissolved in dry CH_2Cl_2 with high dilution (≈0.005 M) under inert atmosphere (nitrogen gas) and Et_3N (0.076 g, 0.759 mmol, 2 equiv), CMPI (0.11 g, 0.43 mmol, 1 equiv) was added at rt. Reaction mixture was stirred for 5h at 55 °C temperature. The reaction mixture was diluted with EtOAc (20 mL) and organic layer was washed with 1 N HCl (10 mL) and saturated aqueous $NaHCO_3$ (10 mL) and brine. The organic layer was dried over Na_2SO_4. The solvent was removed in vacuo to give the crude product that was purified by column chromatography to afford oxazolone pseudotetrapeptide **2a** as a white solid (0.25 g, 67%), R_f = 0.6 (ethyl acetate), $[\alpha]_D^{25}$ = +133.66 (*c* = 0.16, MeOH), Mp = decomposes to >250 °C, ν_{max} (KBr) = 3444 – 3421, 1740, 1688 cm^{-1}.

^1H NMR (500 MHz, $CDCl_3$): δ = 9.03 (s, 1H, NH-1), 8.52 (s, 1H, NH-2), 6.19 (d, *J*= 3.5 Hz, 1H, C_1H^c), 6.07 (d, *J*= 3.6 Hz, 1H, C_1H^b), 6.04 (d, *J*= 3.6 Hz, 1H, C_1H^d), 5.91 (d, *J*= 3.2 Hz, 1H, C_1H^a), 5.29 (d, *J*= 3.6 Hz, 1H, C_2H^b), 5.00 (d, *J*= 3.5 Hz, 1H, C_2H^c), 4.78 (d, *J*= 5.8 Hz, 1H, C_4H^c), 4.77 (d, *J* = 7.5 Hz, 1H, C_4H^d), 4.66 (d, 1H, C_4H^a), 4.65 (d, *J* = 5.4 Hz, 1H, C_4H^b), 4.44 - 4.40 (m, 2H, $C_5H^{a,c}$), 4.35 (d, *J*= 3.6 Hz, 1H, C_2H^d), 4.32 (d, *J*= 3.6 Hz, 1H, C_2H^a), 4.18 - 4.07 (m, 2H, $C_5H^{b,d}$), 4.04-3.90 (m, 8H, $C_6H^{a,b,c,d}$), 1.80 (bs, 2H, NH_2), 1.62-1.22 (m, 48H, CH_3).

^{13}C NMR (125 MHz, $CDCl_3$): δ = 170.8 ($\underline{C}$OONH-), 170.6 ($\underline{C}$OO-), 166.7 ($\underline{C}$OONH-), 163.0 ($\underline{C}$=N), 114.0 ($CH_3\underline{C}OOCH_3$-d), 113.4 ($CH_3\underline{C}OOCH_3$-a), 112.9 ($CH_3\underline{C}OOCH_3$-c), 112.6 ($CH_3\underline{C}OOCH_3$-b), 109.8 ($CH_3\underline{C}OOCH_3$-d), 109.7 ($CH_3\underline{C}OOCH_3$-a), 109.6 ($CH_3\underline{C}OOCH_3$-b), 109.0 ($CH_3\underline{C}OOCH_3$-c), 106.2 (C1-d), 105.8 (C1-c), 105.5 (C1-b), 105.2 (C1-a), 87.6 (C2-a), 86.2 (C2-d), 85.9 (C2-c), 83.2 (C2-b), 82.4 (C4-a), 82.3 (C4-b), 82.0 (C4-c), 81.1 (C4-d), 76.0

(C3-d), 73.9 (C5-a), 73.3 (C5-b), 73.2 (C5-c), 73.1 (C5-d), 70.3 (C3-b), 68.0 (C6-d), 67.9 (C3-a), 67.2 (C3-c), 66.7 (C6-a), 66.4 (C6-b), 66.0 (C6-c), 27.0 (CH_3), 26.84 (CH_3), 26.79 (CH_3), 26.75 (CH_3), 26.72 (CH_3), 26.68 (CH_3), 26.65 (CH_3), 26.5 (CH_3), 26.42 (CH_3), 26.40 (CH_3), 26.3 (CH_3), 26.2 (CH_3), 25.5 (CH_3), 25.3 (CH_3), 25.2 (CH_3), 25.1 (CH_3).

^{15}N NMR (50MHz, $CDCl_3$): δ = 26.2 (<u>N</u>H_2), 112.8 (<u>N</u>H-1), 114.1 (<u>N</u>H-2), 246 (-C=<u>N</u>-). HRMS (ESI$^+$): Calculated for $C_{52}H_{77}N_4O_{24}$ (M+H$^+$) 1141.4927 found 1141.4894.

^{1}H NMR assignment of compound 2a:

Table S1: ^{1}H NMR chemical shift (ppm), coupling constant (*J* Hz) and multiplicity of **2a***

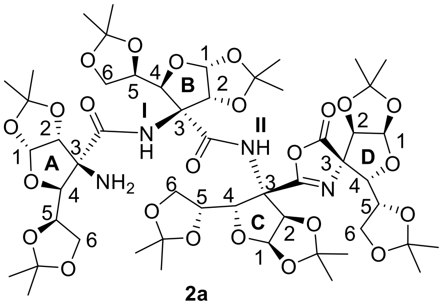

2a

Proton	Chemical Shift (ppm), coupling constant (*J* Hz) and multiplicity			
	Ring A	Ring B	Ring C	Ring D
NH(I)	-	9.03 (s)	-	-
NH(II)	-	-	8.52 (s)	-
H-1	5.91 (d, *J*= 3.6)	6.07 (d, *J*= 3.6)	6.19 (d, *J*= 3.5)	6.04 (d, *J*= 3.6)
H-2	4.32 (d, *J*= 3.6)	5.29 (d, *J*= 3.6)	5.00 (d, *J*= 3.5)	4.35 (d, *J*= 3.6)
H-4	4.66 (d, *J*= 8.6)	4.66 (d, *J*= 5.4)	4.78 (d, *J*= 5.8)	4.78 (d, *J*= 7.5)
H-5	4.44 - 4.40 (m)	4.07 (m)	4.44 - 4.40 (m)	4.07 (m)
Ha-6	4.08 (m)	4.06 (m)	4.16 (*J*= 4.3, 8.9)	4.21 (*J*= 6.1, 8.4)
Hb-6	3.93 (t, *J*= 8.3)	4.06 (m)	4.03 (t, J = 4.3)	4.07 (m)
NH_2	1.80 (bs)	-	-	-

*acetonide CH_3 signals were not shown.

Oxazolone pseudotetrapeptide acetate **2b**:

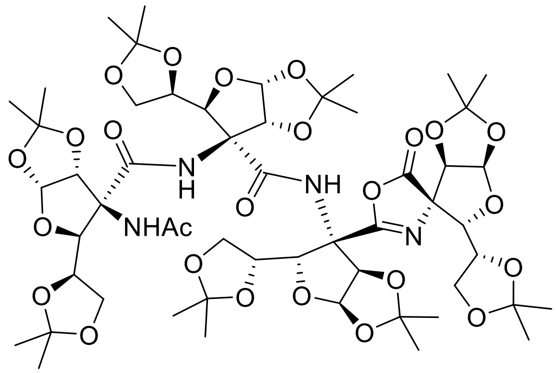

To a mixture of **2a** (0.04 g, 0.034 mmol) and pyridine (0.0056 mL, 0.068 mmol) in DCM (3 mL) at 0 ^{o}C under nitrogen atmosphere was added acetic anhydride (0.034 g, 0.035 mmol) and the reaction was allowed to attain room temperature and stirred for 12 hours. With the completion of reaction, the solvent was removed under reduced pressure to give the crude product that was purified by column chromatography to afford N-acetylated oxazolone pseudotetrapeptide **2b** as a white solid (0.034 g, 84%), R_f = 0.5 (ethyl acetate), $[\alpha]_D^{25}$ = +126.43 (c = 0.16, MeOH), IR ν_{max} (KBr) = 3372, 3309, 2986, 2934,1709, 1686 cm^{-1}.

^{1}H NMR (500 MHz, CDCl$_3$): δ 8.24 (s, 1H), 8.19 (s, 1H), 8.09 (s, 1H), 6.19 (d, J = 3.8 Hz, 1H), 6.06 (d, J = 3.6 Hz, 1H), 5.99 (d, J = 3.5 Hz, 1H), 5.92 (d, J = 3.6 Hz, 1H), 5.30 (d, J = 3.8 Hz, 1H), 5.14 (d, J = 3.5 Hz, 1H), 5.00 (d, J = 3.4 Hz, 1H), 4.67 (d, J = 9.2 Hz, 1H), 4.59 (dd, J = 11.0, 4.5 Hz, 1H), 4.48 (d, J = 2.6 Hz, 1H), 4.41 (d, J = 6.7 Hz, 2H), 4.35 – 4.29 (m, 3H), 2.10 (s, 3H), 1.67 – 1.52 (m, 23H), 1.44 – 1.21 (m, 40H).

^{13}C NMR (125 MHz, CDCl$_3$): δ = 171.6, 170.9, 167.5, 165.0, 164.1, 114.1, 113.5, 113.4, 112.3, 109.9, 109.7, 109.5, 109.4, 107.5, 106.5, 105.2, 104.0, 85.7, 85.6, 83.7 (C2-b), 83.4 (C4-a), 83.0, 81.5, 73.1, 73.07, 72.38, 69.84, 69.42, 68.45, 67.74, 67.17, 66.27, 65.47, 66.0 , 26.95, 26.83, 26.75, 26.68, 26.58, 26.46, 26.41, 26.37, 26.12, 25.64, 25.27, 25.09, 23.75.HRMS (ESI^{+}): Calculated for $C_{54}H_{78}N_4O_{25}$ (M+H^{+}) 1182.4927 found 1205.4822.

^{1}H and ^{13}C NMR spectra:

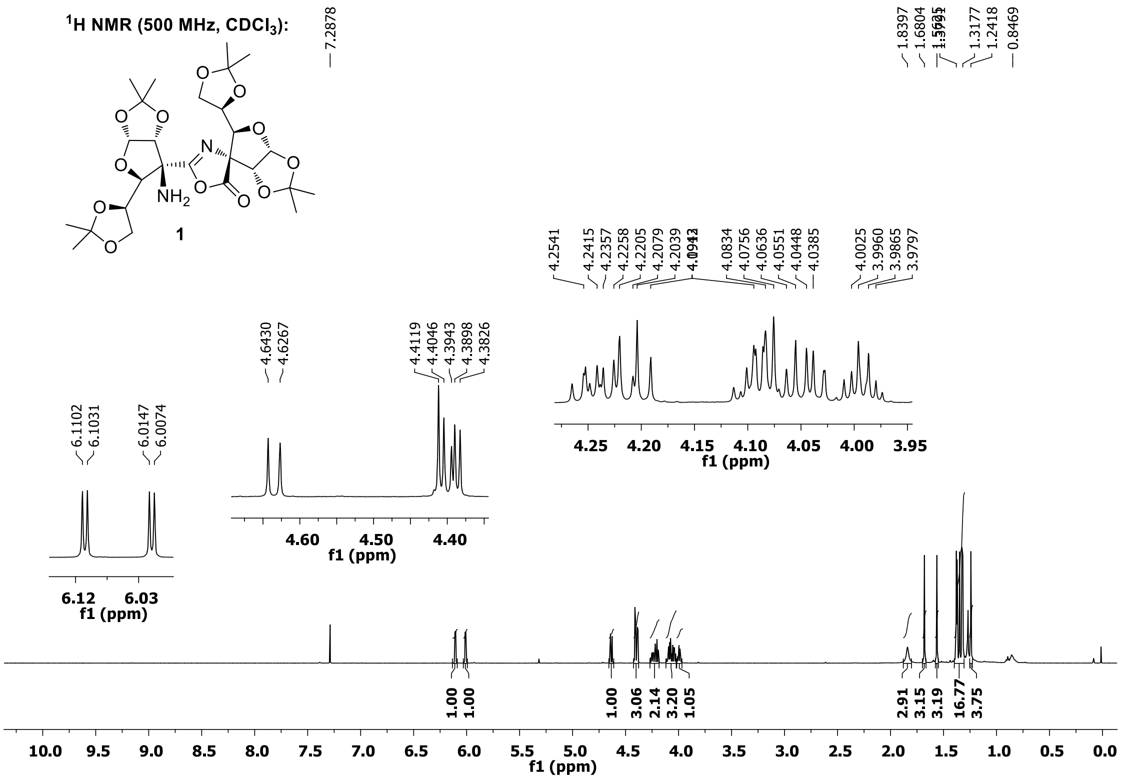

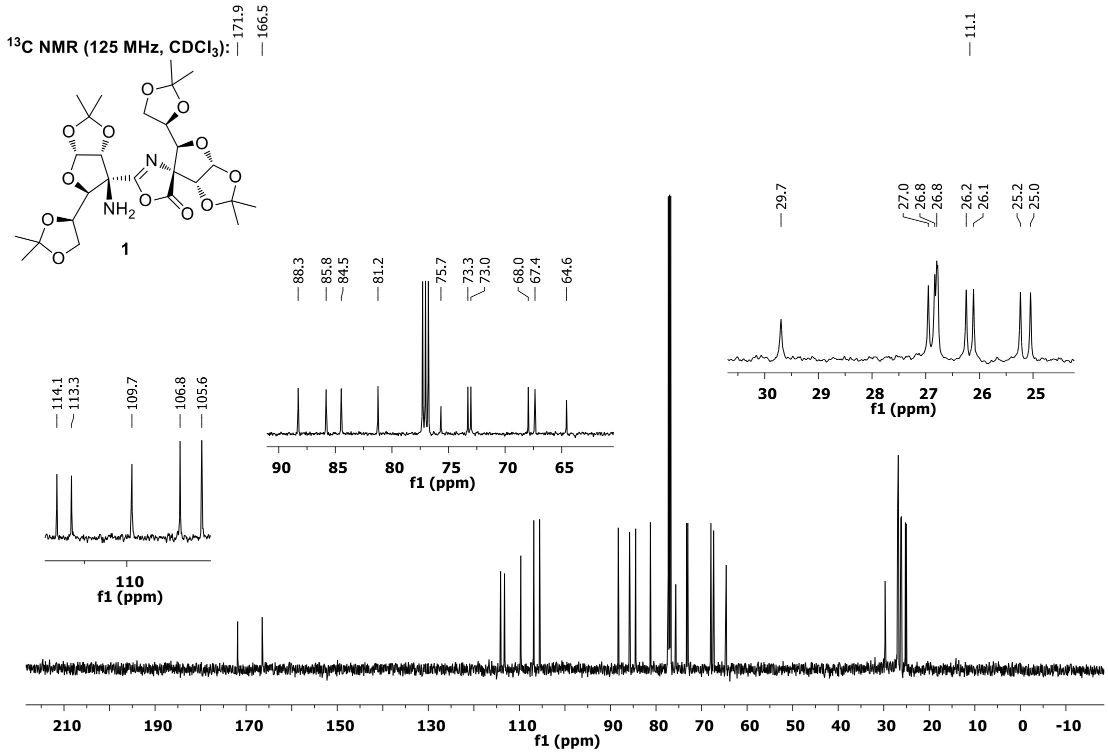

Figure S1: ^{1}H and ^{13}C NMR of compound **1**.

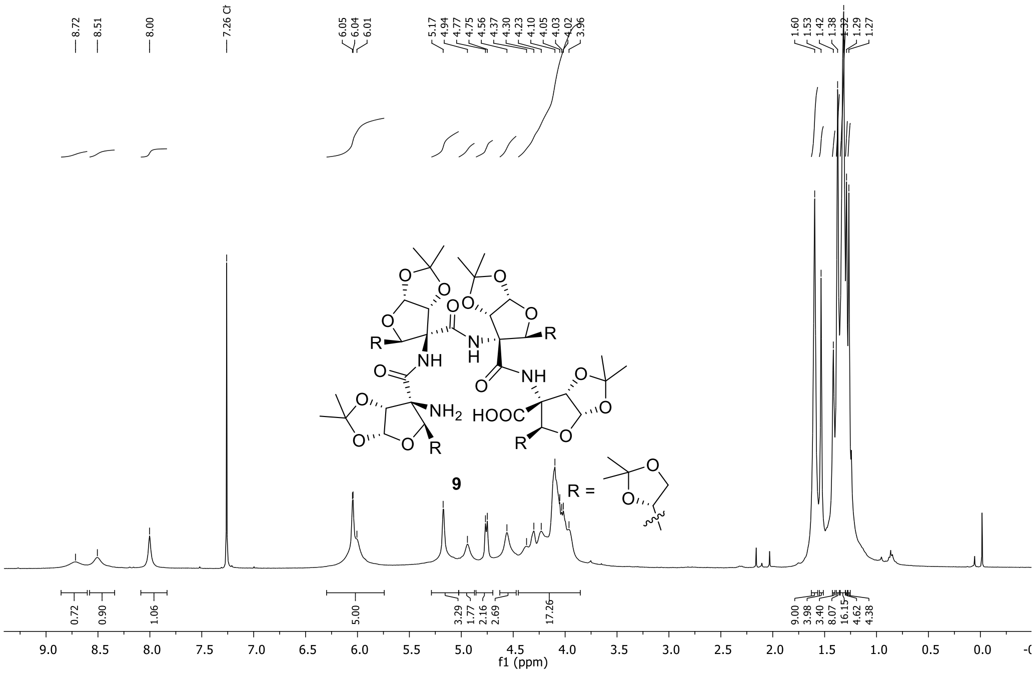

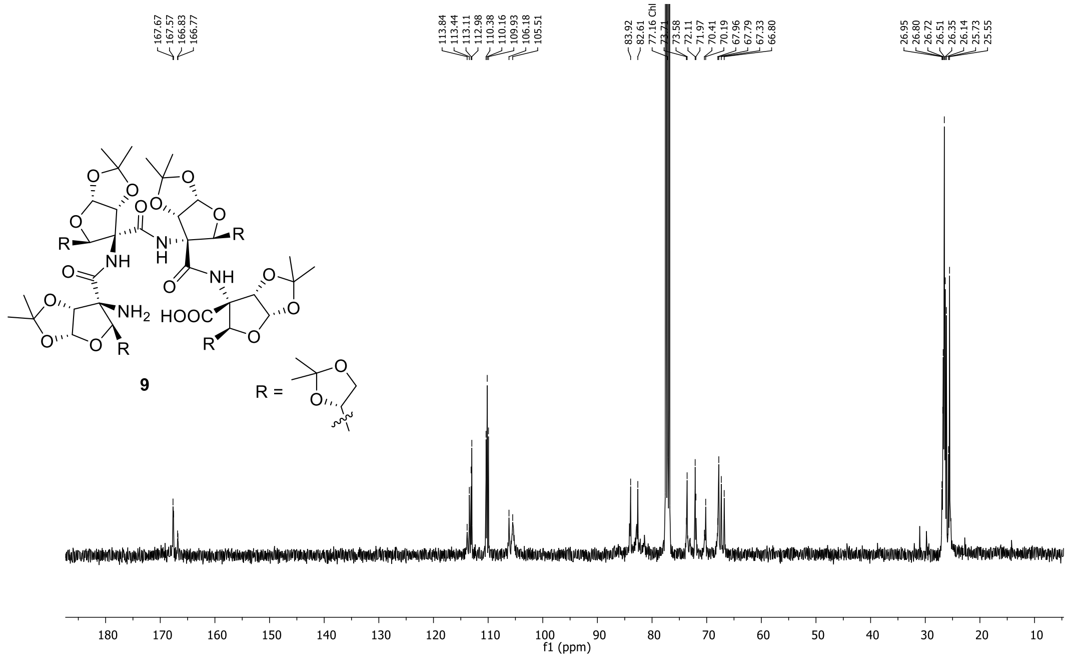

Figure S2: ^{1}H and ^{13}C spectra of compound **9**

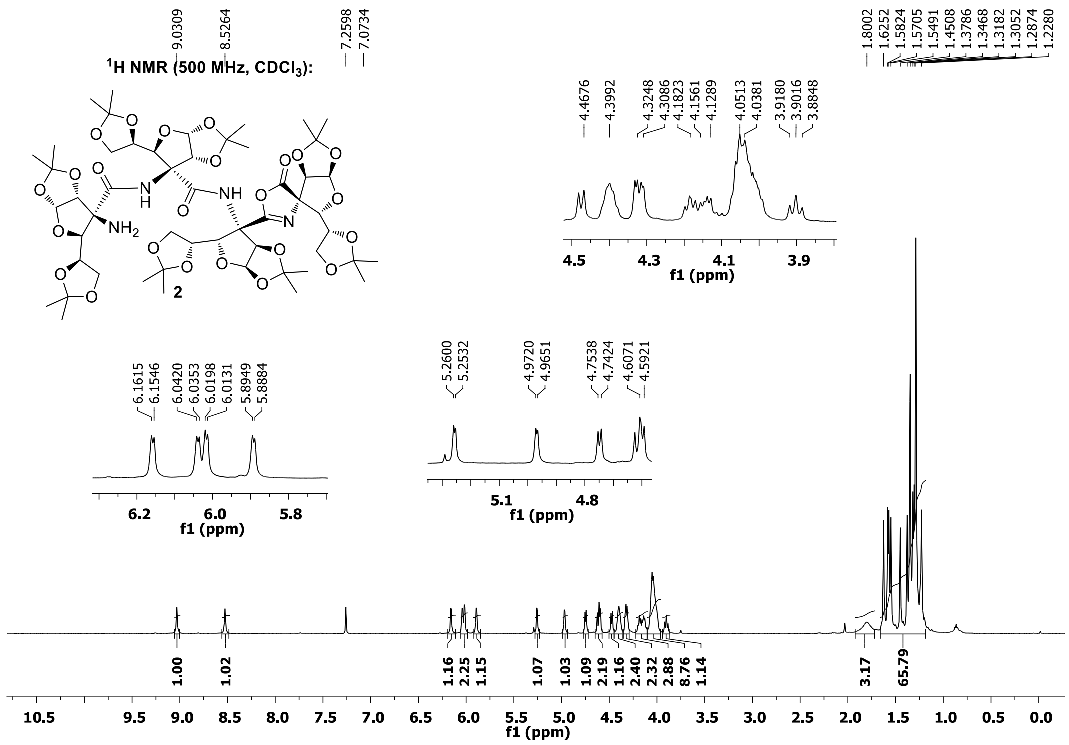

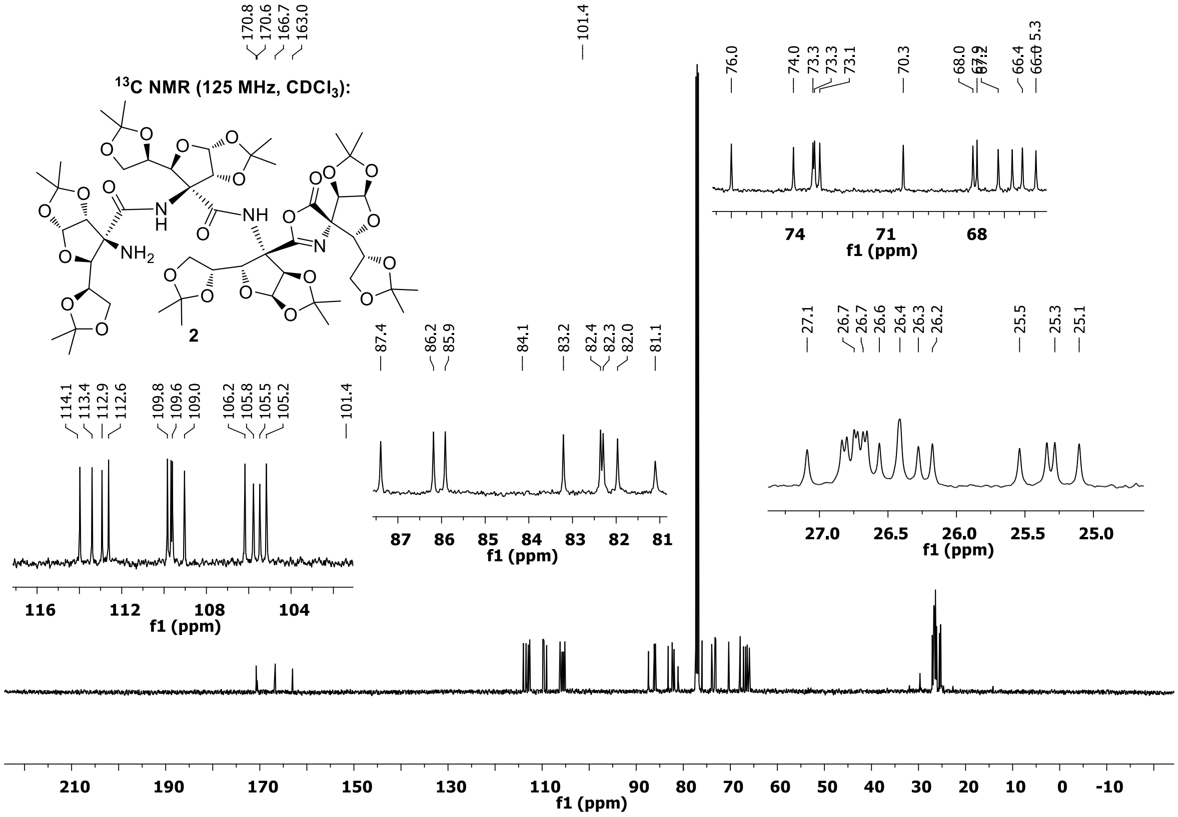

Figure S3: ^{1}H and ^{13}C NMR of compound **2a**.

Compound 2b. ^{1}**H NMR (500 MHz, CDCl$_3$):**

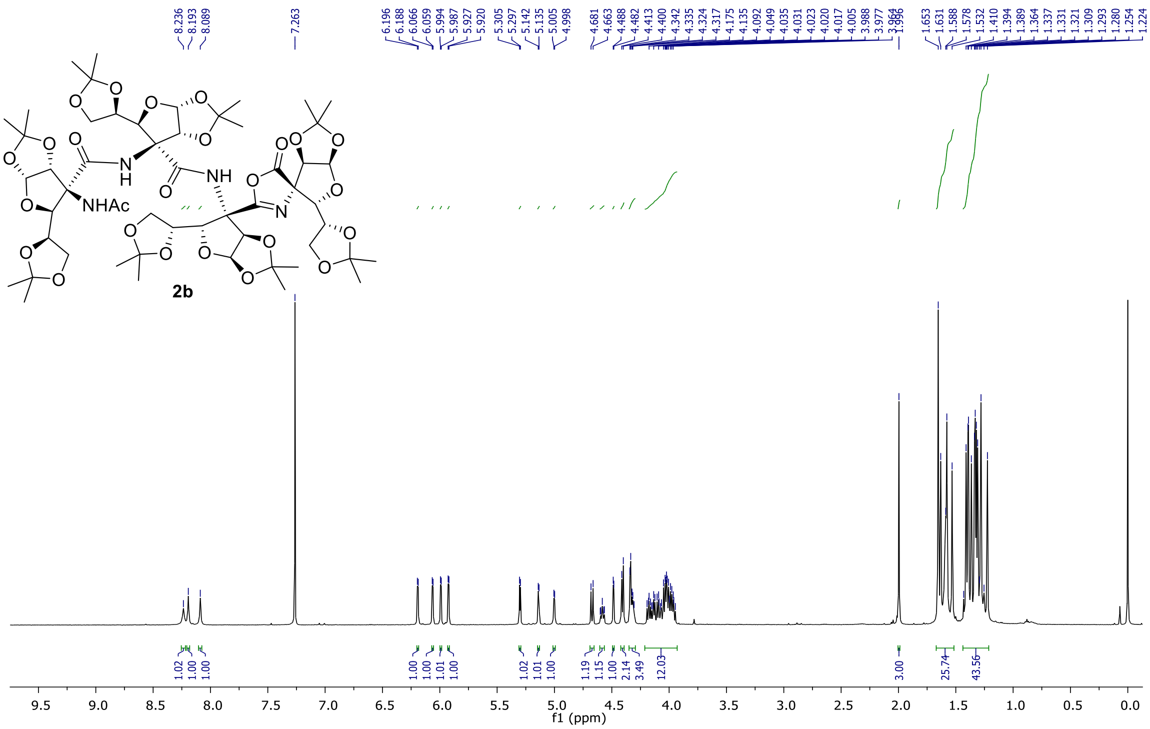

Compound 2b. ^{13}**C NMR (125 MHz, CDCl$_3$):**

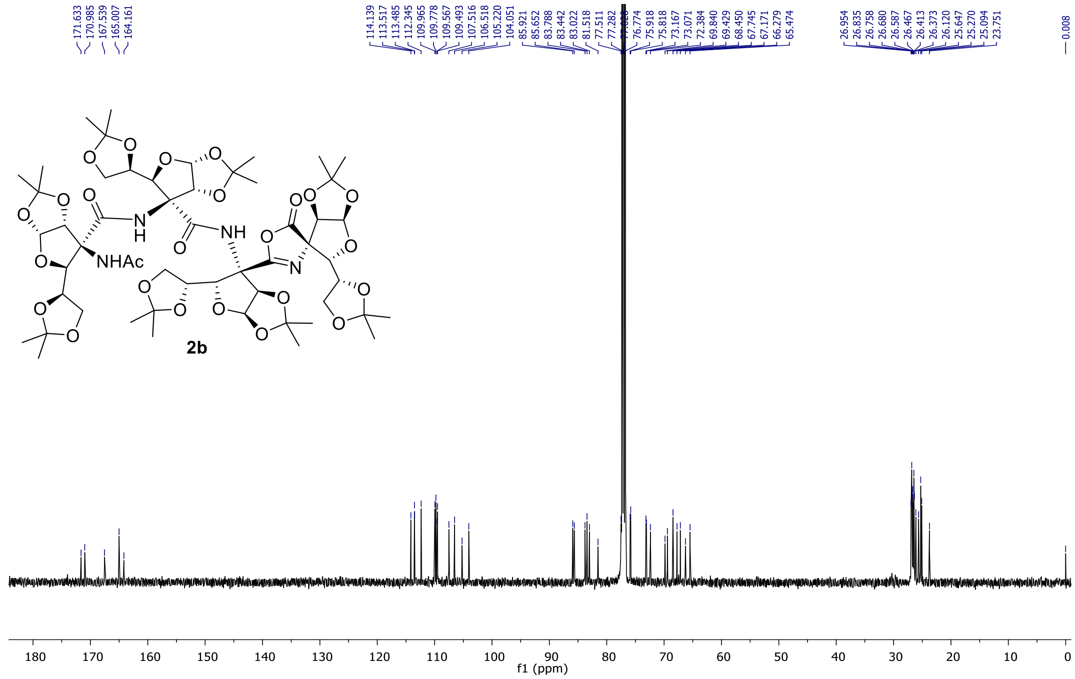

Compound 2b. DEPT (125 MHz, $CDCl_3$):

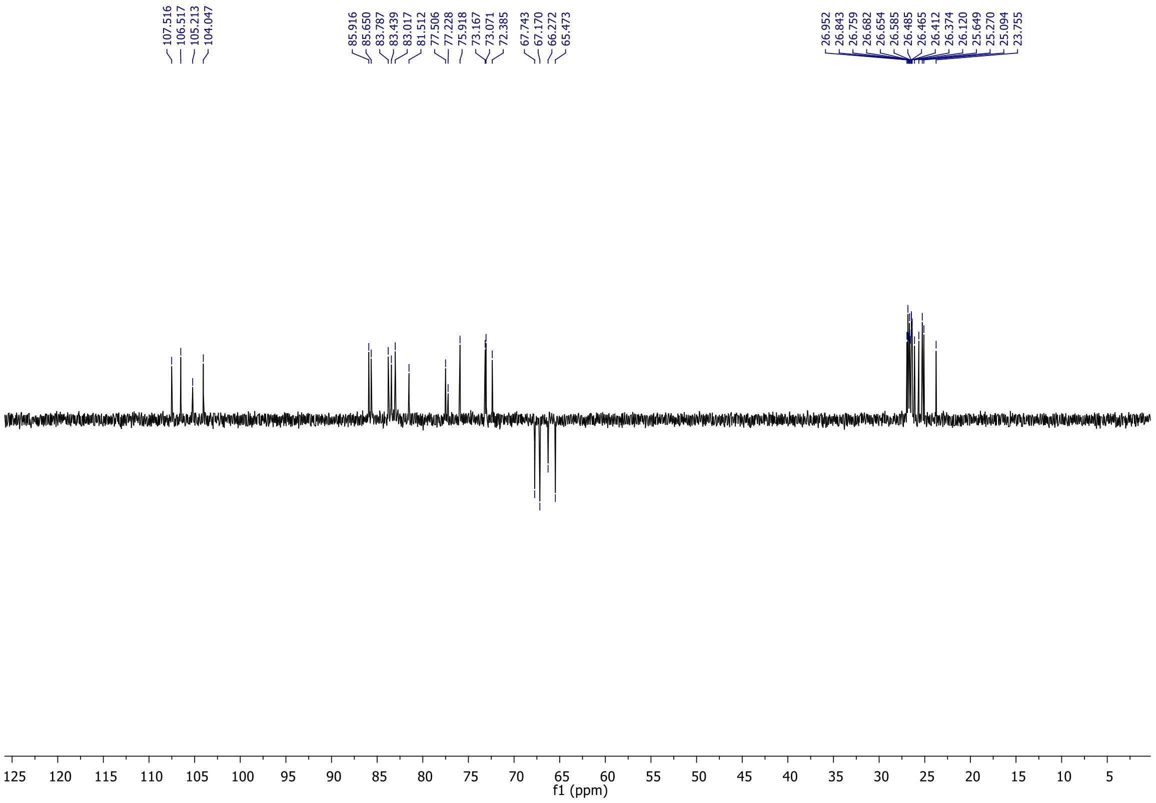

Figure S4: ^{1}H, ^{13}C and DEPT NMR of compound **2b**.

2D-NMR spectra of 2a:

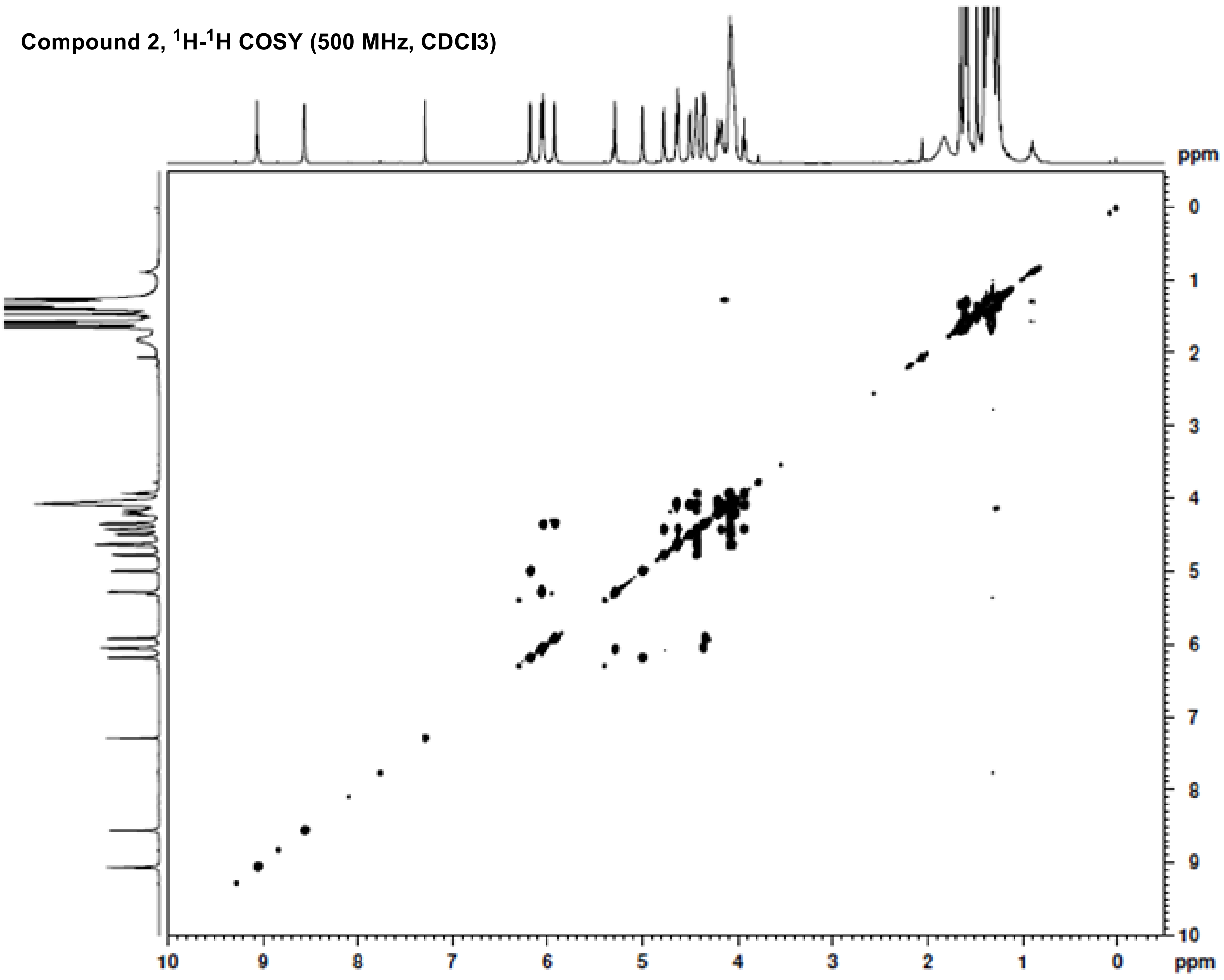

Figure S5: ^{1}H-^{1}H COSY spectra of compound **2a**.

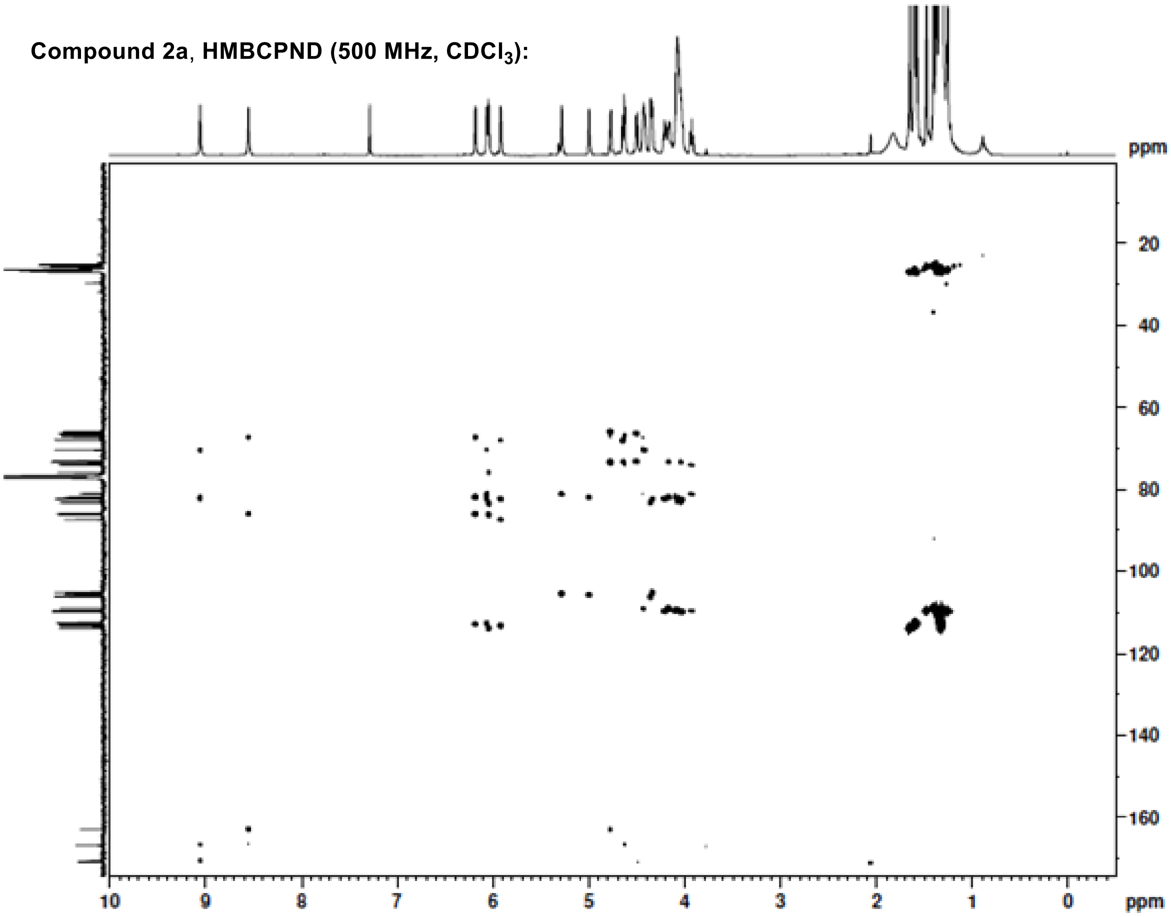

Figure S6: ^{1}H-^{13}C HMBC spectra of compound **2a**.

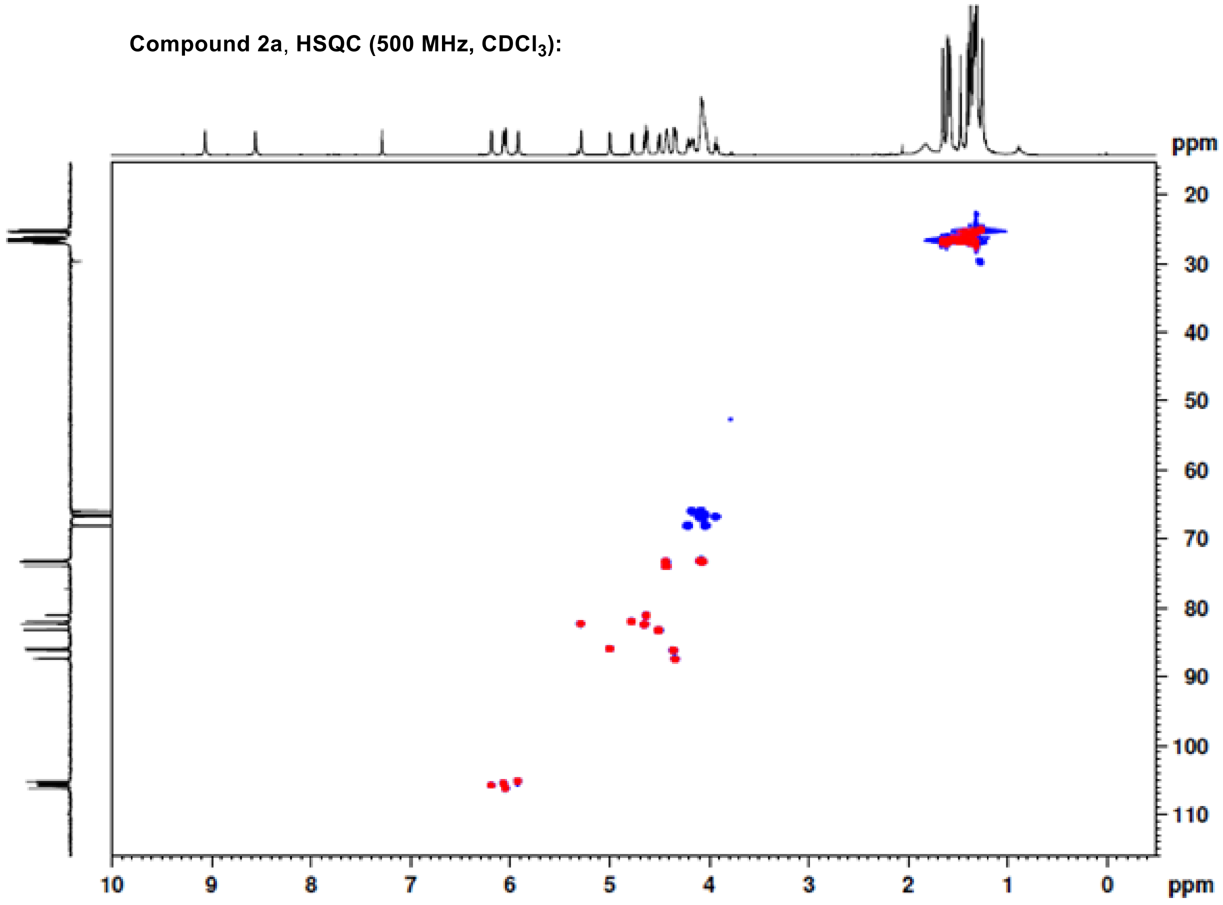

Figure S7: HSQC spectra of compound **2a**.

Compound 2a, NOESY (500 MHz, $CDCl_3$):

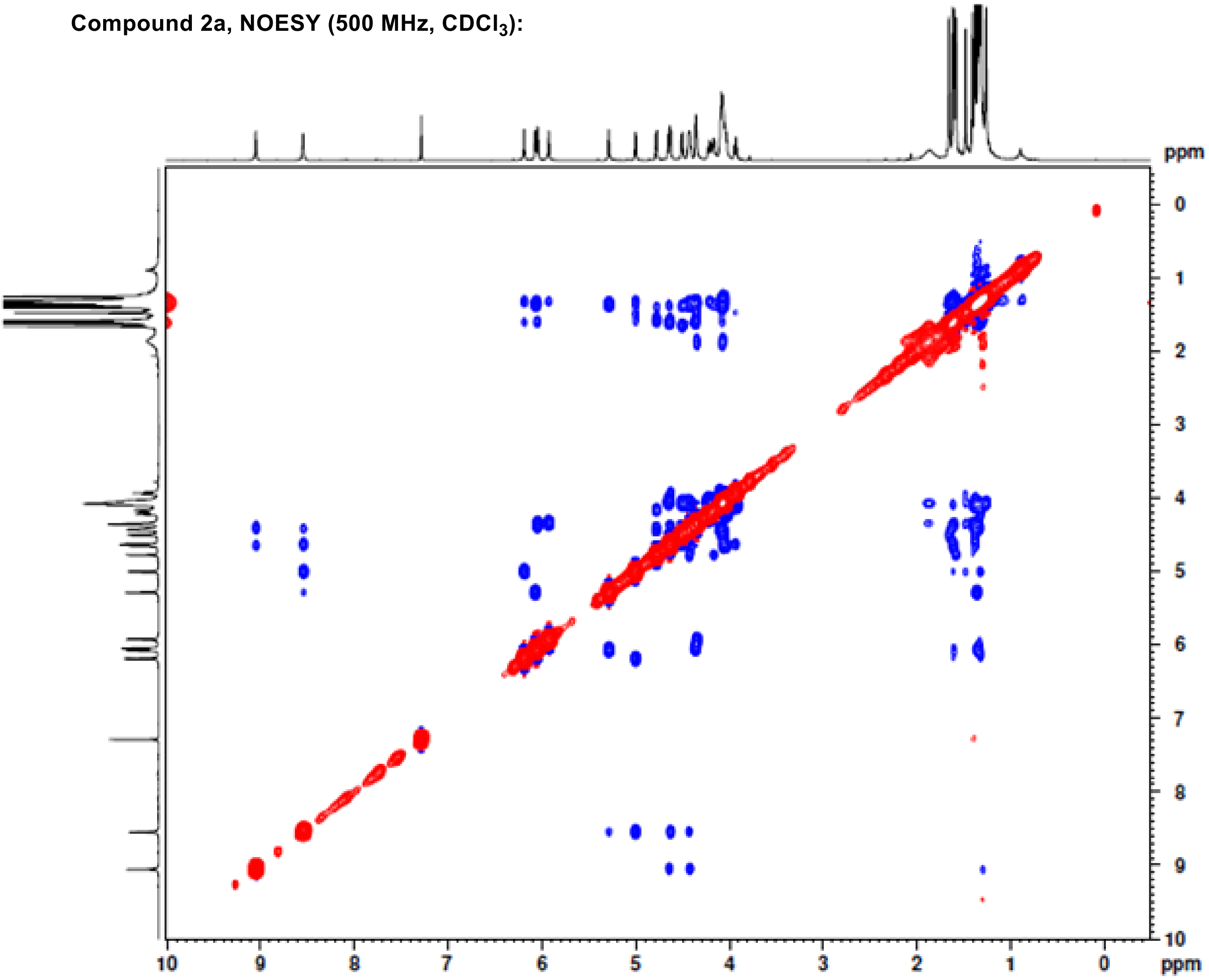

Figure S8: NOESY spectra of compound **2a**.

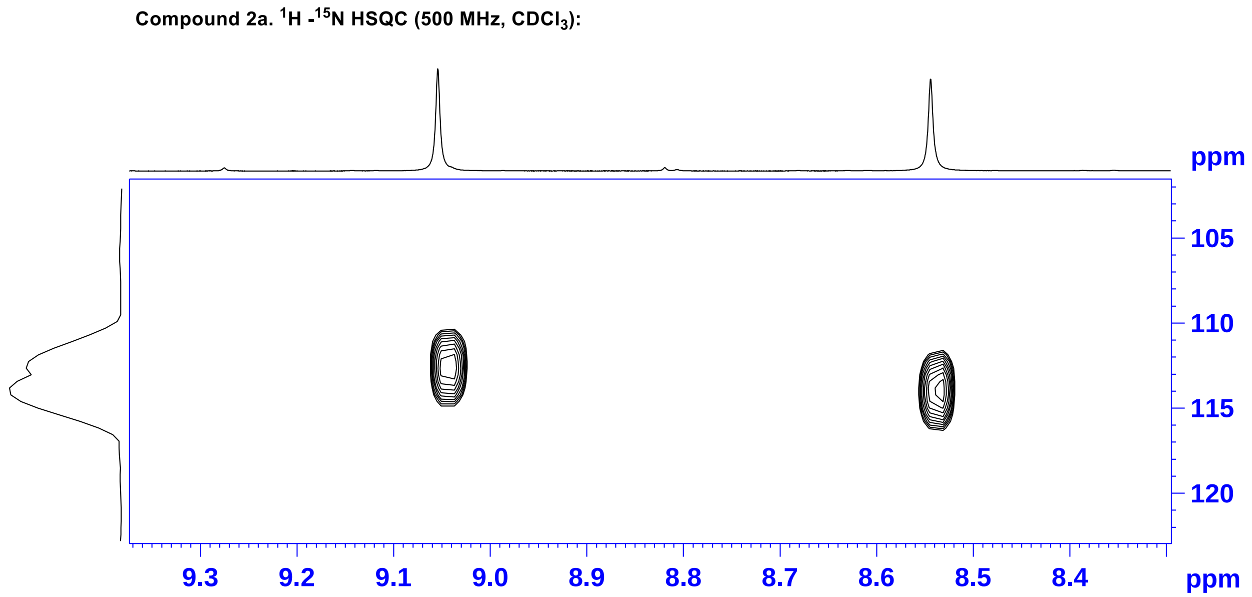

Figure S9: ^{1}H-^{15}N HSQC spectra of compound **2a**.

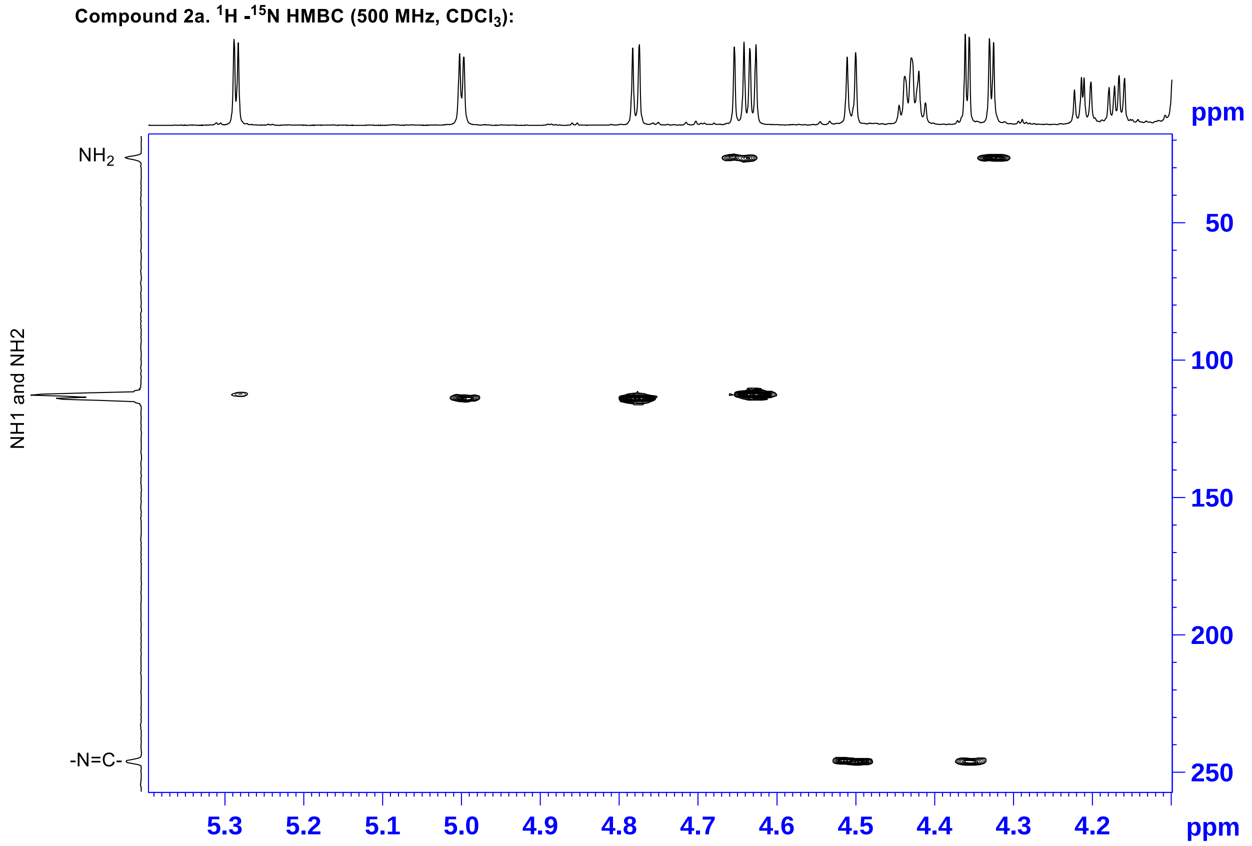

Figure S10: ^{1}H-^{15}N HMBC spectra of compound **2a**.

Ion transport activity studies:

Buffer and stock solution preparation: HEPES buffer was prepared by dissolving solid HEPES (10 mM) and NaCl (100 mM) in autoclaved water, followed by adjustment of pH (pH = 7.0) by adding NaOH solution. Stock solution of all transporter forming molecules was prepared in HPLC grade DMSO.

Preparation of EYPC-LUVs⊃HPTS: The vesicle preparation was performed according to the known literature procedure.[1] A thin transparent film of egg yolk phosphatidylcholine (EYPC) was prepared by drying of 1 mL of EYPC (25 mg/mL in $CHCl_3$) with purging of nitrogen and continuous rotation in a clean and dry small round-bottomed flask. The transparent film was kept in high vacuum for 3 h to remove all trace of $CHCl_3$. The resulting films were hydrated with 1 mL buffer (1 mM HPTS, 10 mM HEPES, 100 mM NaCl, pH = 7.0) for 1 h with 4–5 times vortexing occasionally and subjected to a freeze-thaw cycle (≥15 times). Extrusions were done 19 times (must be an odd number) by a Mini-extruder with a polycarbonate membrane, pore size 100 nm (Avanti). Extravesicular dyes were removed by gel filtration (Sephadex G-50) with buffer (10 mM HEPES, 100 mM NaCl, pH = 7.0) and diluted to 6 mL to get EYPC-LUVs⊃HPTS: ≈5.0 mM EYPC, inside: 1 mM HPTS, 10 mM HEPES, 100 mM NaCl, pH = 7.0, outside: 10 mM HEPES, 100 mM NaCl, pH = 7.0.

Ion transport activity experiment: In a clean and dry fluorescence cuvette equipped with magnetic stirrer, EYPC-LUVs⊃HPTS suspension 25 µL was added in buffer solution (HEPES 10 mM, NaCl 100 mM, pH = 7.0). The cuvette was placed in fluorescence instrument (Fluoromax-4 from Jobin Yvon Edison) and allowed stir under slow condition by magnetic stirrer equipped with the instrument. The fluorescence emission intensity of HPTS dye was monitored as time course at λ_{em} = 510 nm (λ_{ex} = 450 nm). The pH gradient (ΔpH ≈ 0.8) was created by addition of 20 µL 0.5 M NaOH solution in extravesicular buffer at t = 20 s. The transporter molecule of required concentration was added at t = 100 s as a DMSO solution. Finally the vesicles were lysed with addition of 25 µL 10% Triton-X 100 to get the complete destruction of pH gradient. For data analysis and comparison, using Equation 1, the fluorescence emission intensity (Y-axis) was normalized between the point of transporter addition (i.e. I_F at t = 100 s was normalized to I_F = 0) and end point of experiment (i.e. I_F at t = 350 s was normalized to I_F = 100) (Figure S11). Then the time of transporter addition was adjusted to t = 0 s.

$I_F = [(F_t - F_0) / (F_\infty - F_0)] \times 100$ Equation 1.

Where, I_F = normalized fluorescence intensity, F_0 = Fluorescence intensity just before the channel forming molecule addition (at 0 s). F_∞ = Fluorescence intensity at saturation after complete leakage (at 320 s). F_t = Fluorescence intensity at time t.

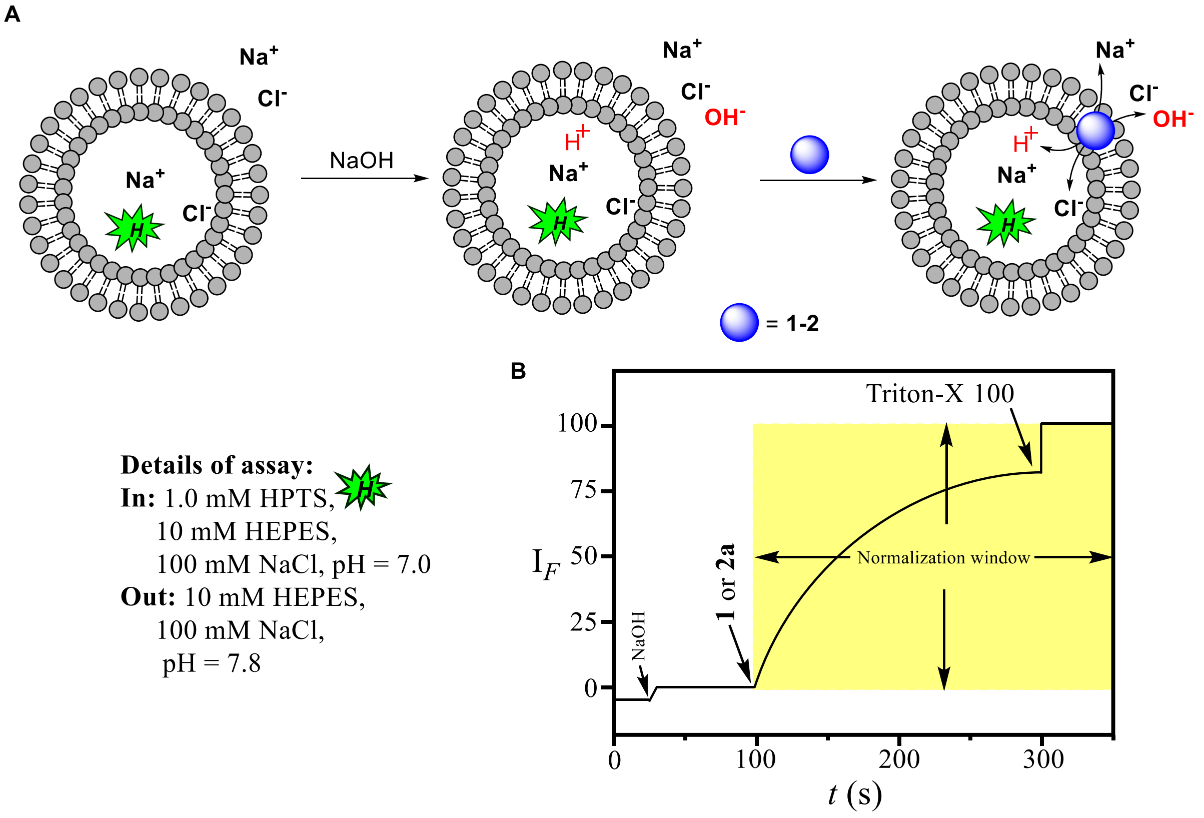

Figure S11: Representative fluorescence-based ion transport activity across EYPC-LUVs⊃HPTS (**A**), Illustration of ion transport activity showing normalization window (**B**).

The concentration profile data were analyzed by Hill equation (Equation S2) to get the effective concentration (EC_{50}):

$Y = Y_\infty + (Y_0 - Y_\infty) / [1 + (c / EC_{50})^n]$ Equation S2

where, Y = fractional fluorescence intensity, Y_0 = fluorescence intensity just before the channel forming compound addition (at t = 0 s), Y_∞ = fluorescence intensity with excess compound concentration, c = concentration of channel forming compound.

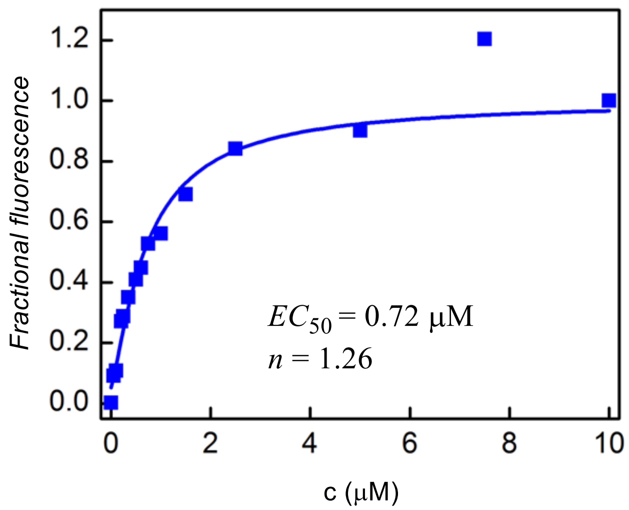

Figure S12: Hill plot of **2a**, and EC_{50} value obtained from Hill analysis.

Ion selectivity studies:

Buffer and stock solution preparation: HEPES buffer was prepared by dissolving solid HEPES (10 mM) and appropriate salt (100 mM) in autoclaved water, followed by adjustment of pH (pH = 7.0) by adding NaOH solution. Stock solutions of all transporter molecules were prepared in HPLC grade DMSO.

Preparation of EYPC-LUVs⊃HPTS: A thin transparent film of egg yolk phosphatidylcholine (EYPC) was prepared by drying of 1 mL of EYPC (25 mg/mL in $CHCl_3$) with purging of nitrogen and continuous rotation in a clean and dry small round-bottomed flask. The transparent film was kept in high vacuum for 3 h to remove all trace of $CHCl_3$. The resulting films were hydrated with 1 ml buffer (1 mM HPTS, 10 mM HEPES, 100 mM NaCl, pH = 7.0) for 1 h with 4–5 times vortexing occasionally and subjected to freeze-thaw cycle (≥15 times). Extrusions were done 19 times (must be an odd number) by a Mini-extruder with a polycarbonate membrane, pore size 100 nm (Avanti). Extravesicular dyes were removed by gel filtration (Sephadex G-50) with buffer (10 mM HEPES, pH = 7.0) and diluted to 6 mL to get EYPC-LUVs⊃HPTS: ≈5.0 mM EYPC, inside: 1 mM HPTS, 10 mM HEPES, 100 mM NaCl, pH = 7.0, outside: 10 mM HEPES, pH = 7.0.

Ion selectivity assay: In a clean and dry fluorescence cuvette 1975 µL of HEPES buffer (10 mM HEPES, 100 mM M^+X^- (where, M^+ is an alkali metal cation and X^- is a monovalent inorganic anion), pH = 7.0) was added followed by addition of 25 µL of EYPCLUVs⊃HPTS in slowly stirring condition by a magnetic stirrer equipped with the fluorescence instrument (at t = 0 s).

The time course of HPTS fluorescence emission intensity, Ft was observed at λ_{em} = 510 nm (λ_{ex} = 450 nm). 20 µL of 0.5 M NaOH was added to the cuvette at t = 20 s to make the pH gradient between the intra and extra vesicular system. Transporter compound **2a** were added at t = 100 s and at t = 300 s, of 10% Triton X-100 (25 µL) was added to lyse vesicles for the complete destruction of pH gradient. All fluorescence time-dependent spectra were normalized between 0 (at 96 s) and 100 s (at 320 seconds) by using Equation S1. Then the time of transporter addition was adjusted to t = 0 s.

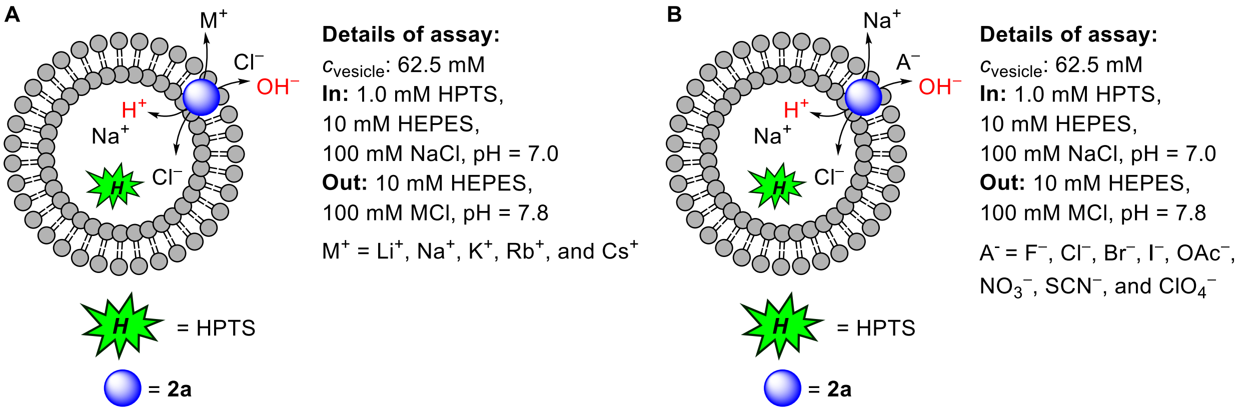

Figure S13: Schematic representations of the fluorescence based cation (**A**) and anion (**B**) selectivity assays.

Cation selectivity was checked by varying the extravesicular chloride salts (MCl) of different alkali metal cations (Fig. S13A). The difference in rate observed for different cation (Li^+, Na^+, K^+, Rb^+, Cs^+) is characteristics of cation selectivity of Ion transporter. Anion selectivity was evaluated by varying the extravesicular sodium salts of different halides (NaX) (Figure S13B). In these assays, NaCl, NaBr, and NaI were used. The difference in transport activity was observed due to the competitive transport of X^-/OH^-.

Ion transport activity by Lucigenin assay:

Preparation of EYPC-LUVs⊃Lucigenin for concentration dependent assay and symport assay: A solution (1 mL) of EYPC lipid (25 mg/mL) lipid dissolved in $CHCl_3$ was taken in a clean and dry small round bottom flask. The solvents were evaporated slowly by a stream of nitrogen, followed by drying under vacuum for at least 5 h. After that 1 mL of 1 mM N,N'-dimethyl-9,9′-biacridinium dinitrate (lucigenin) in 200 mM $NaNO_3$ (dissolved in 10 mM Hepes

buffer with pH=7.0) was added, and the suspension was hydrated for 1 h with occasional vortexing of 4-5 times and then subjected to freeze-thaw cycle (≥ 15 times). The vesicle solution was extruded through a polycarbonate membrane with 200 nm pores for minimum 19 times (must be an odd number), to give vesicles with a mean diameter of ≈200 nm. The extracellular lucigenin was removed from the vesicles by size exclusion column chromatography (Sephadex G-50) using 200 mM $NaNO_3$ as eluent. The obtained vesicles were diluted to 4 mL with 200 mM $NaNO_3$.

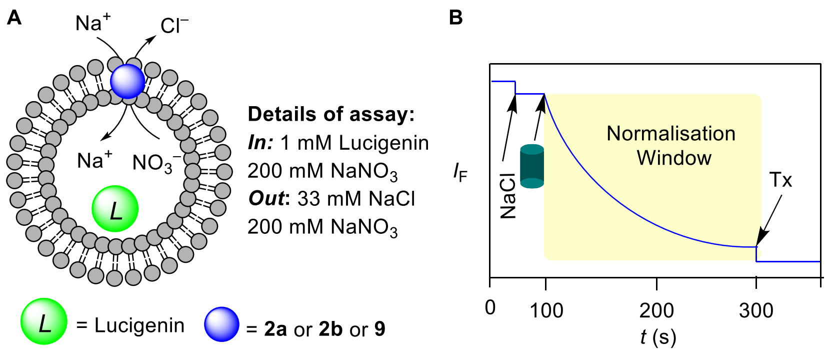

Figure S14. Schematic representation of chloride influx assay using EYPC-LUVs⊃Lucigenin (**A**) and representative fluorescence kinetics experiment of corresponding assay (**B**).

Chloride influx assay: In clean and dry fluorescence cuvette, 200 mM $NaNO_3$ (1975 µL) and EYPC-LUVs⊃lucigenin (25 µL) were taken. This suspension was placed in a slowly stirring condition in fluorescence instrument equipped with a magnetic stirrer (at t = 0 s). The fluorescence intensity of lucigenin was monitored at λ_{em} = 535 nm (λ_{ex} = 455 nm) as a course of time. The chloride gradient was created by the addition of 2.0 M NaCl (33.3 µL) at t = 20 s between intra- and extravesicular system, followed by the addition of transporter at t = 100 s. Finally, vesicles were lysed by addition of 10% Triton X-100 (25 µL) at t = 300 s for the complete destruction of chloride gradient.

The time-dependent data were normalized to percent change in fluorescence intensity using Equation S3:

$$I_F = [(I_t - I_0) / (I_\infty - I_0)] \times (-100) \qquad \text{Equation S3}$$

where, I_0 is the initial intensity, I_t is the intensity at time t, and I_∞ is the final intensity after addition of Triton X-100. Then the time of transporter addition was adjusted to t = 0 s.

Cation selectivity assay across EYPC-LUVs⊃lucigenin vesicles: The vesicles were prepared by following the same protocol as stated above. In clean and dry fluorescence cuvette, 200 mM $NaNO_3$ (1975 μL) and EYPC-LUVs⊃lucigenin (25 μL) were taken. The suspension was kept in slowly stirring condition in fluorescence instrument equipped with a magnetic stirrer at t = 0 s. The quenching of fluorescence intensity of lucigenin was monitored as a course of time at λ_{em} = 535 nm (λ_{ex} = 455 nm). At t = 20 s, the chloride gradient was created by addition of 2 M chloride salts (33.3 μL) of different cations MCl (M = Li^+, Na^+, K^+, Rb^+, and Cs^+), followed by addition of transporter **2a** at t = 100 s. Finally, vesicles were lysed by the addition of 10% Triton X-100 (25 μL) at t = 300 s to destruct the applied chloride gradient completely. The time-dependent data were normalized to percent change in fluorescence intensity using Equation S3. Then the time of transporter addition was adjusted to t = 0 s.

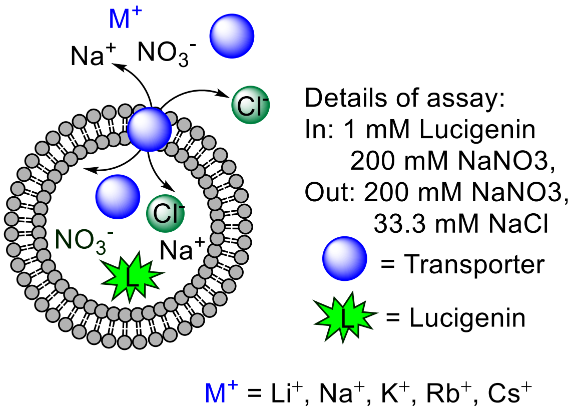

Figure S15. Schematic representations of fluorescence-based cation selectivity assay.

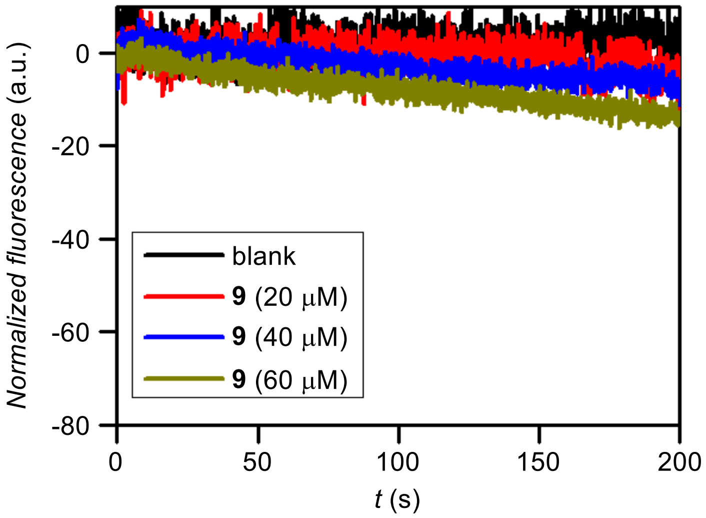

Figure S16: Concentration dependent activity of **9** across EYPC-LUVs⊃lucigenin.

Ion transport activity across EYPC-LUVs⊃lucigenin in the presence of valinomycin: The antiport mechanism (i.e., simultaneous transport of two different ions, in opposite directions, across the membrane) of **2a** was experimentally confirmed by lucigenin assay in the presence of valinomycin. The ion transport activity of **2a** was monitored in vesicle entrapped with lucigenin (1 mM) and $NaNO_3$ (200 mM) suspended in $NaNO_3$ (200 mM), and a KCl (33 mM) was added. Subsequently, the ion transport was monitored after the addition of the transporter **2a** (20 µM). For monitoring the ion transport in the presence of valinomycin, valinomycin (1 µM) was added prior to the addition of the transporter **2a**.

The remarkable enhancement in ion transport activity of **2a** in the presence of valinomycin gave a direct experimental insight of antiport mechanism of ion transport.

Preparation of EYPC-LUVs⊃lucigenin: The vesicles were prepared by the following protocol as stated above.

Antiport mechanism: In clean and dry fluorescence cuvette 1975 µL 200 mM $NaNO_3$ solution and 25 µL EYPC-LUVs⊃lucigenin vesicles were taken and slowly stirred in fluorescence instrument equipped with a magnetic stirrer (at t = 0 s). The time-dependent fluorescence intensity of lucigenin was monitored at λ_{em} = 535 nm (λ_{ex} = 455 nm). A solution of 2 M KCl (33.3 µL) was added at t = 20 s to create chloride gradient between intra and extra vesicular system, followed by the addition of valinomycin (1 µM) at t = 50 s and transporter **2a (**10 µM) at

t = 100 s. Finally, destruction of chloride gradient was done by addition of 10% Triton X-100 (25 μL) at *t* = 300 s. The time-dependent data were normalized to percent change in fluorescence intensity using Equation S3. Then the time of transporter addition was adjusted to t = 0 s.

Dilution studies of 2a in $CDCl_3$:

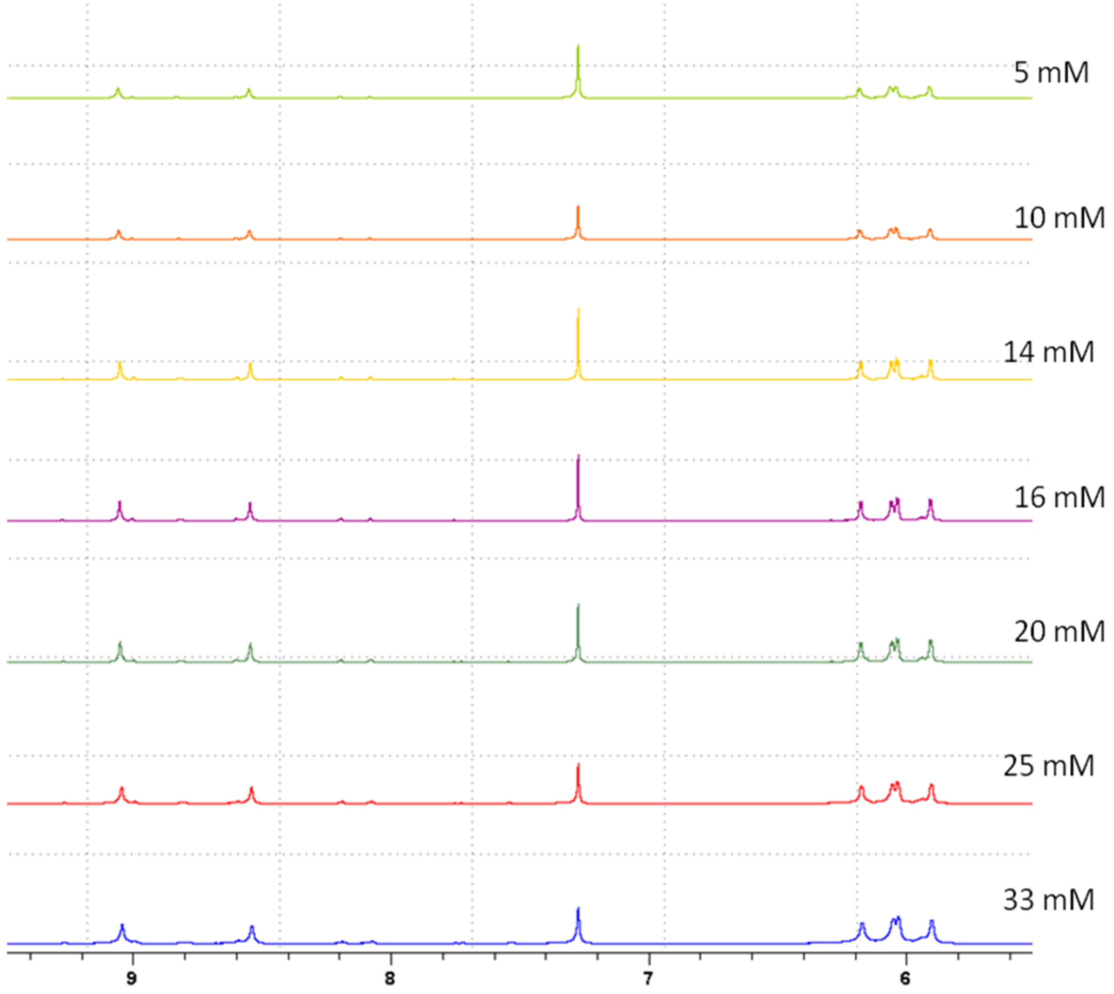

Figure S17: Partial ^{1}H NMR (500 MHz) of **2** in $CDCl_3$ with different concentrations.

Geometrically optimized models of 2a, 2b and 9:

The geometrically optimized models of **2a, 2b** and **9** showed small structural changes with respect to helical pitch length. The distance between C=O···N(II) is 3.18 Å in **2a,** 3.29 Å in **2b** and 3.43 Å in **9**. The distance between $C\alpha_1 \cdots C\alpha_4$ is 9.67 Å in **2a**, 9.64 Å in **2b** and 9.84 Å in **9**.

Similarly, the distance between N1···C4 is 9.44 Å in **2a**, 9.51 Å in **2b** and 10.47 Å in **9**. This suggests that compound **9** is slightly elongated helix than compound **2a** and **2b** which supports the compact helical architecture for **2a** and **2b** due to the presence of oxazolone ring leading to γ-turn conformation as shown in Figure **18**.

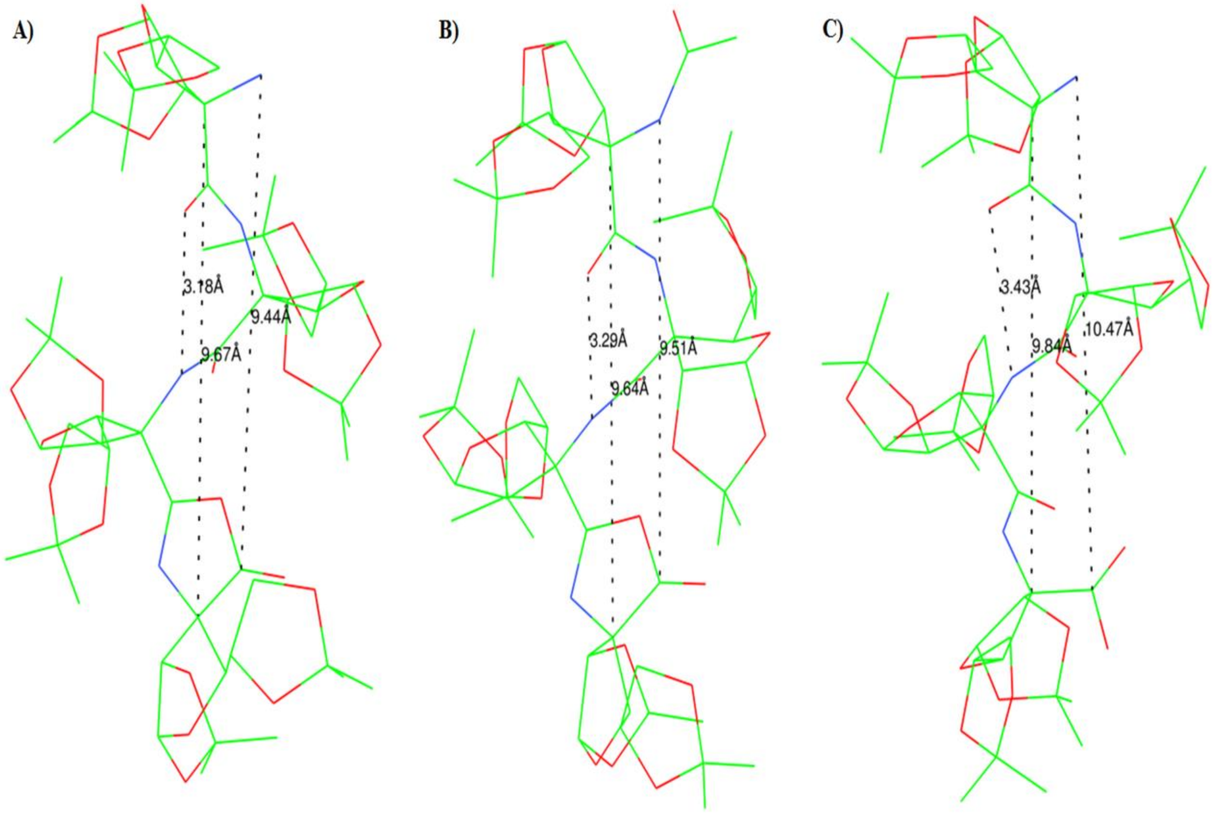

Figure S18: Geometrically optimized models of **2a**, **2b** and **9** with distance measurements.

References:

1 Vangala, M.; Dhokale, S. A; Gawade, R. L.; Rajamohanan, R. P.; Puranik V. G.; Dhavale, D. D.; *Org. Biomol. Chem*. **2013**, *11*, 6874.

2 a) Forman, G. S; Scaffidi A.; Stick, R. V.; *Aust. J. Chem*. **2004**, *57*, 25; b) Scaffidi, A.; Skelton, B. W.; Stick, R. V.; White, A. H.; *Aust. J. Chem*., **2007**, *60*, 93; c) Scaffidi, A.; Skelton, B. W.; Stick, R. V.; White, A. H.; *Aust. J. Chem*., **2004**, *57*, 733.

3 (a) Talukdar, P.; Bollot, G.; Mareda, J.; Sakai, N.; Matile, S.; *J. Am. Chem. Soc.* 2005, **127**, 6528. (b) Gorteau, V.; Bollot, G.; Mareda, J.; Perez-Velasco A.; Matile, S.; *J. Am. Chem. Soc.* 2006, **128**, 14788. (c) Saha, T.; Hossain, M. S.; Saha, D.; Lahiri, M.; Talukdar, P.; *J. Am. Chem. Soc.* 2016**, 138**, 7558. (d) Burade, S. S.; Shinde, S. V.; Bhuma, N.; Kumbhar, N.; Kotmale, A.; Rajmohanan, P. R.; Gonnade, R. G.; Talukdar, P.; Dhavale. D. D.; *J. Org. Chem*. 2017, **82**, 5826. (e) Burade, S. S.; Saha, T.; Bhuma, N.; Kumbhar, N.; Kotmale, A.; Rajamohanan, P. R.; Gonnade, R. G.; Talukdar, P.; Dhavale, D. D.; *Org. Lett*. 2017, **19**, 5948.
